# A case study on new high-strength temporary support technology of extremely soft coal seam roadway

**DOI:** 10.1038/s41598-023-48583-7

**Published:** 2023-12-04

**Authors:** Zhijun Xu, Chong Li, Yue Cao, Lianhai Tai, Jun Han

**Affiliations:** 1 School of Mining Engineering, China University of Mining and Technology, Xuzhou, China; 2 Changzhou BYD Auto Co., Chang Zhou, China

**Keywords:** Coal, Civil engineering

## Abstract

One of the main challenges in excavating roadways is implementing temporary supports that are powered by hydraulics and have high strength. The current temporary support system lacks active support and often causes separation between the top plate and the layer below. It is crucial to control the initial separation of the roadway roof for the stability of the surrounding rock, especially on roadways with loose and soft rock. This research focuses on the A4027 return airway in Sail Six Mine. The issues with the temporary support system in this airway have been identified. The concept and principle of using hydraulically driven, high-strength temporary support technology are proposed. A mechanical analysis model is created to study the stacked roof in the temporary support region, and the critical conditions for delamination of the top plate are determined. The relationship between the delamination difficulty parameter Q, the distance between temporary supports L, and the strength of the temporary supports q is quantified. Numerical simulation using Flac3d is used to model the relationship between the strength of the temporary supports and the deformation and stress of the rock on the roof. The overall strength of the temporary supports for the A4027 return airway is determined to be 10 kN/m^2^, with a distance of 2 m between the temporary supports. Hydraulically driven, high-strength temporary support devices are developed and tested for their strength. Field trials are conducted as well. The results show that the initial separation of the top plate is improved and that the support effect in the temporary support region is significant. The maximum separation of the top plate during excavation is only 34 mm, and the sinking of the top plate does not exceed 68 mm. This effectively limits the deformation of the surrounding rocks in the very soft coal seam, providing valuable insights for other roadways with similar conditions.

## Introduction

The front explorer beam is currently the most popular temporary support technique used in underground excavation work. However, this technique has certain limitations that need to be addressed. Firstly, it cannot provide an active support force on its own, which means that it cannot guarantee worker safety in roof control areas. Secondly, it requires strong manual labor, making it physically demanding for workers. Additionally, it cannot effectively control the maintenance of top plate separation during the early stages of excavation. Furthermore, the front explorer beam technique fails to fully understand the true purpose of temporary support, especially during the excavation process. Therefore, it is crucial to examine the relationship between the strength and length of the temporary support and the stress release and deformation of the nearby rock roof. By investigating this relationship, we can develop a more effective and efficient temporary support technique. To address these issues, a hydraulically driven, high-strength temporary support technique has been proposed^1–4^. This technique aims to overcome the limitations of the front explorer beam by providing an active support force, ensuring worker safety, reducing the need for manual labor, and effectively controlling the maintenance of top plate separation. Through further research and development, this new technique has the potential to greatly improve the safety and efficiency of underground excavation work.

In recent years, numerous academics have investigated the issue of temporary support strength and temporary empty roof control using various techniques such as theoretical analysis, numerical simulation, and others^5–9^. Currently, several primary temporary support techniques are being used, including the front-probe beam, point column type, monolithic column + plate beam, machine-loaded type, and self-moving temporary support^10–13^. For different burial depths, Xiao et al.^14^ examined the quantitative link between temporary support and active support. Yanbin et al.^15^ utilized a sequential excavation approach to simulate the deformation law and mechanical properties of temporary support in a soil roadway, and they advocated for the installation of longitudinal connection reinforcement between each steel frame and steel structure. Shuhui et al.^16^ presented a temporary support system that can adapt to complicated geological constraints and uneven roadway ceilings while ensuring the coordinated functioning of multiple pieces of equipment. The mechanical qualities of the self-moving temporary support (SmTS) were studied during design to meet the demands of the wall rock support system at the mining face^17^. Wang et al. adopted MIDAS finite element software to analyze the influence effect and deformation characteristics of temporary steel support when excavating a roadway using a two-step center diaphragm method (CDM)^17^. Li et al.^18^ developed a new type of machine-mounted temporary support to address the problems associated with temporary support using a foreboding bar. Zhang^19^ conducted a finite element analysis (FEA) on the overall structure and key bearing components of temporary support under different working conditions according to industry standards. Li^20^ carried out an FEA on the temporary support equipment under the roof fall condition of a rectangular roadway using ANSYS Workbench and verified that it met the support requirements. Yang^21^ performed an FEA on the roof beam and base of an inchworm-type roadway temporary support, studying the stress and strain characteristics of the roof beam under normal working conditions and the most dangerous working conditions. Li^22^ developed a kind of temporary support matched with the EBZ160 road header that could work under low roof conditions and solved the problem of the poor adaptability of the temporary support to a low-roof roadway.

In conclusion, the equipment and methods utilized for temporary support structures play a critical role in ensuring the stability of temporary roofs in underground excavation projects. In this study, we have examined the instability of the digging face and identified the factors that cause delamination and deformation in the loose-soft coal lane of the Sair Six Mine. We have also determined the conditions that lead to delamination and developed a mechanical analysis model for the stacked top plate in the temporary support area. Additionally, we have established a quantitative relationship between the strength of temporary support, the distance of support, and the difficulty parameter of delamination. We have also explored the connection between temporary support strength and numerical simulation, using simulation to analyze the stress and deformation of the roof slab in the roadway based on the strength and length of the temporary support. The proposed method of using hydraulic-driven, high-strength temporary support not only effectively addresses the issue of temporary support and initial offset of the roof slab in the extremely soft coal seam roadway of Sel6 Mine but also provides guidance and reference for supporting and excavating roadways in similar mine conditions throughout the country.

## Engineering background

### Engineering geological condition

**Figure 1:**
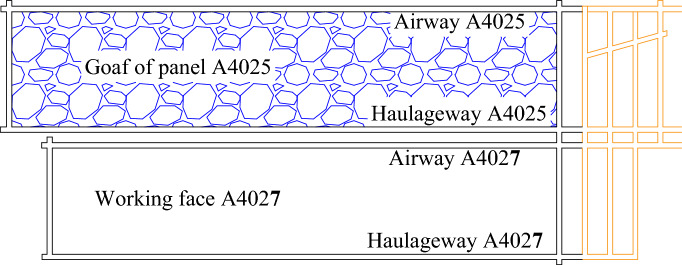
Working face and roadway layout.

As shown in Fig. 1, the A4027 return airway is situated at the + 815 m level in the western section of the second mining area. It is bordered by the A4025 haulageway on the east, the A4027 haulageway on the west, the A4027 cut-hole on the north, and the A4 three-system lane on the south. The thickness of the coal column between the A4025 haulageway and the A4027 return airway is approximately 11–12 m.

**Figure 2:**
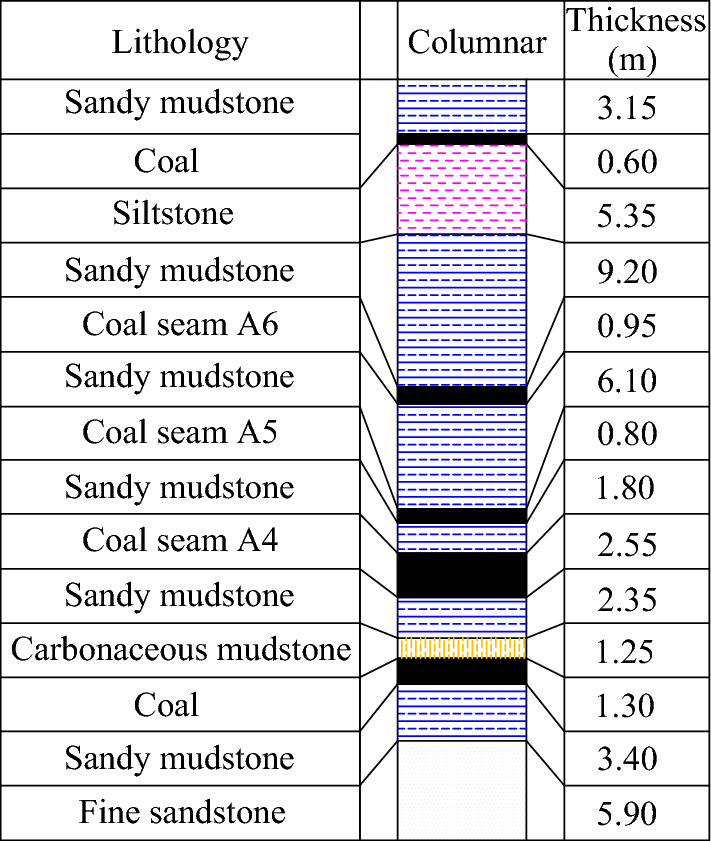
Histogram.

In this area, the average thickness of the coal is 2.55 m, and the coal seam has a dip angle of 12 degrees and a hardness value (f) of 0.7. Above the A4027 return airway, there is a layer of sandstone with an average thickness of 1.5 m. This is followed by a layer of sandstone mudstone with an average thickness of 6.10 m, which forms the uppermost part of the airway. Figure 2, presented as a column diagram, depicts the predominant lithology of the upper plate, which consists of a relatively brittle interlayer of mud and sand.

### The digging face’s deformation and damage characteristics

**Figure 3:**
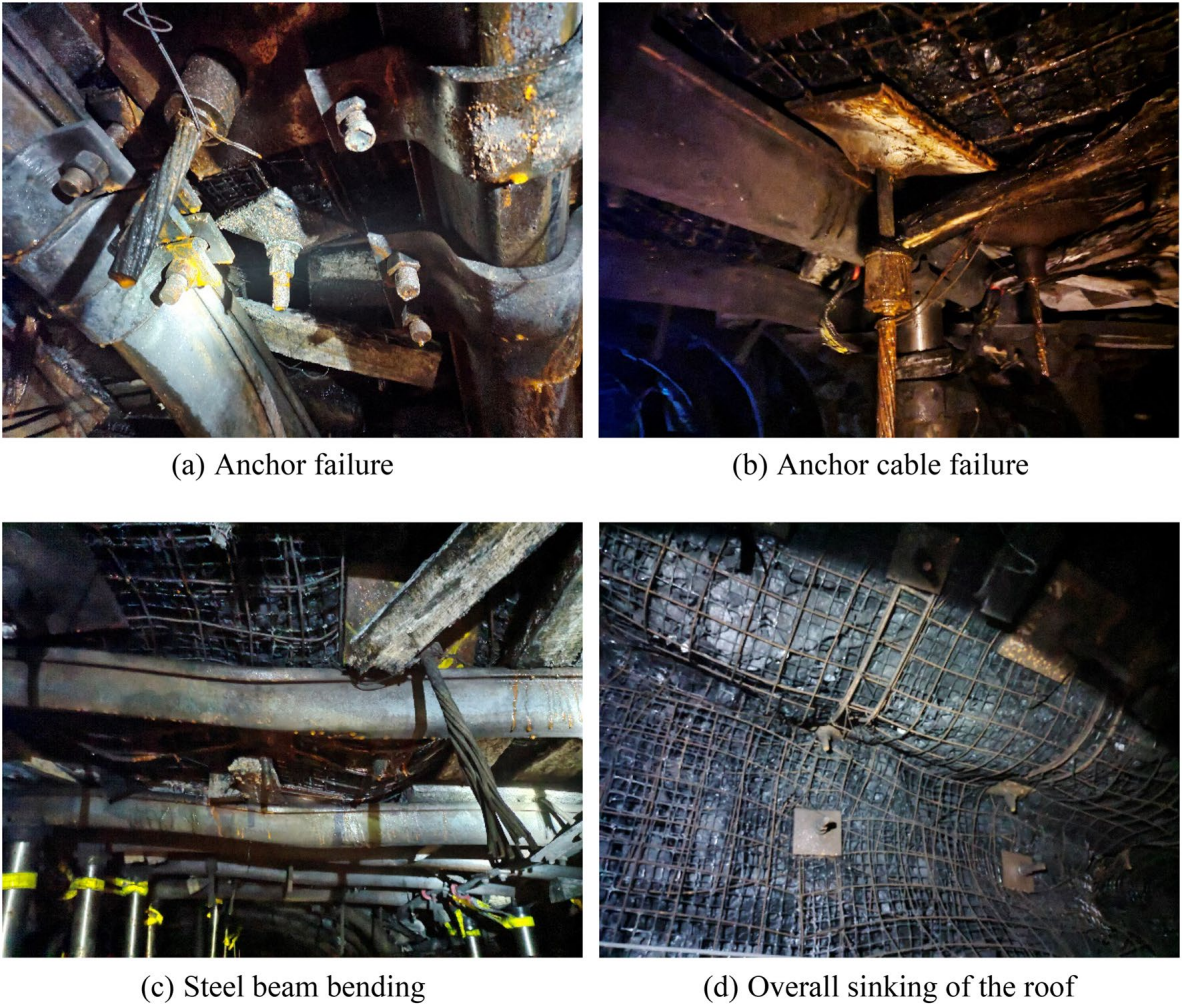
Deformation and damage characteristics of the roadway surrounding rock.

The primary purpose of the front explorer beam is to provide temporary support for the newly excavated roadway’s ceiling. However, the weak surrounding rock in the roadway makes the roof of the temporary support area vulnerable to initial delamination. This can lead to deformation and damage during the later stages of roadway excavation. The main issues observed are anchor failure, anchor cable failure, bending of steel beams, and overall sinking of the roof. These issues are illustrated in Fig. 3.

**Figure 4:**
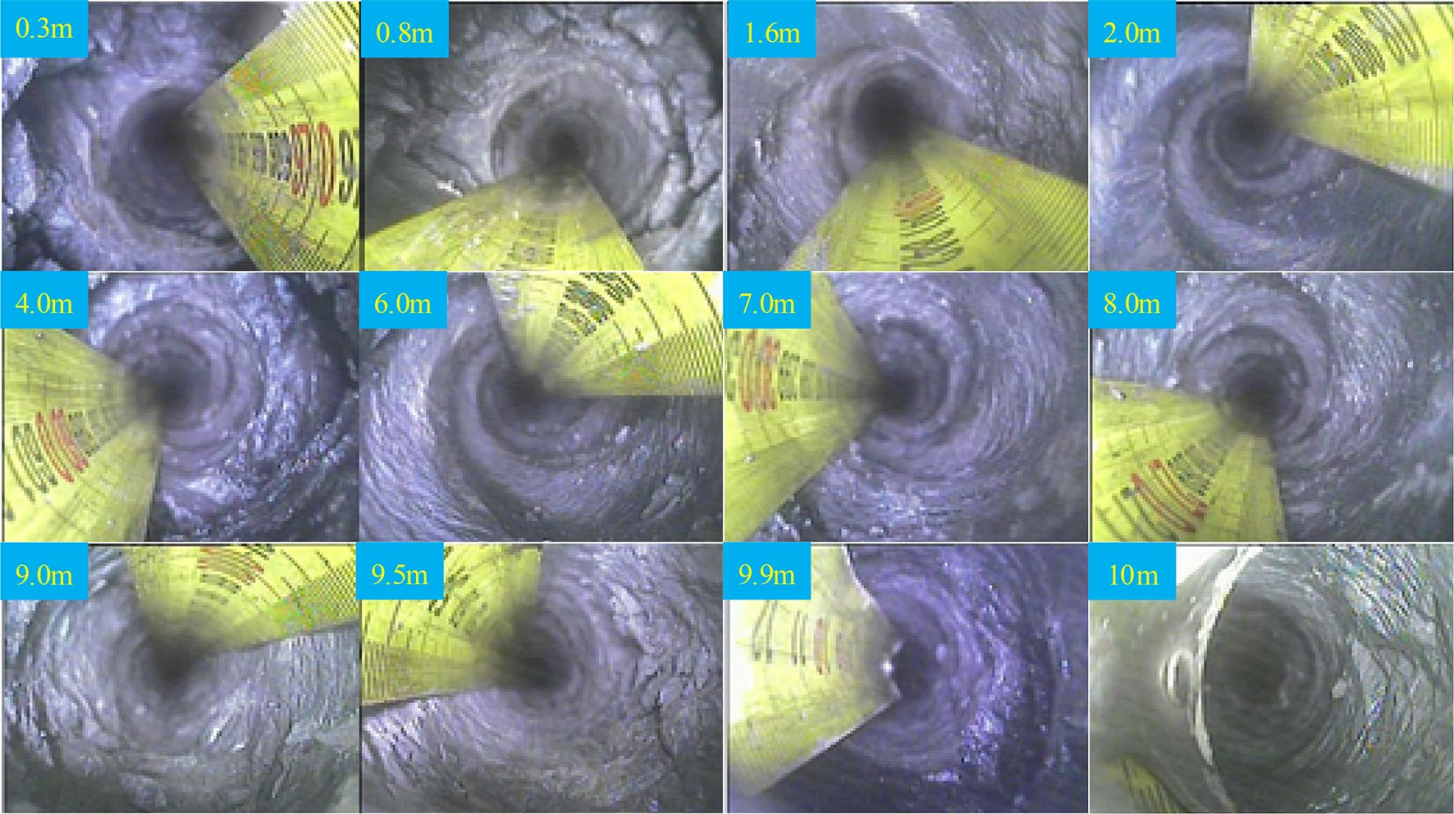
Borehole peeping.

Figure 4 presents the findings from the examination of the top slab in the mine using an electronic drilling peeper. The peeping results reveal that the surrounding rocks of the A4027 return airway exhibit softness, the presence of fissures, and significant modification. The roof of the roadway consists of sandy mudstone in the shallow and central sections, while the deeper sections are composed of siltstone. Furthermore, the structural integrity of the surrounding rocks is poor, and the anchor (cable) support area is located within a weak laminated mudification structure.

### Analysis of the factors that led to the surrounding rock’s deformation at the digging face

Based on the findings mentioned above, it can be concluded that the deformation of the surrounding rock during roadway excavation is caused by two main factors. Firstly, the rock fragmentation strength is low, which leads to the instability of the rock. Secondly, the current temporary support device in place is unable to provide sufficient support to the roof of the roadway, resulting in significant movement of the surrounding rock during the initial excavation. Additionally, the lack of maintenance and inhibition of the top plate worsens the deformation and damage to the surrounding rock in later stages of construction.

### High-strength temporary support technology and method with hydraulic drive

The primary goals of the hydraulically powered, high-strength temporary support are twofold. Firstly, it aims to establish a mechanical model of the roof in the temporary support area to study the relationship between the strength of the temporary support (q) and the distance of the temporary support (L). This analysis also explores how the strength (q) and distance (L) of the temporary support hinder the initial delamination of the roof. Secondly, it aims to investigate the distribution of anchor pre-stress bearing area at the separation of rows. Ultimately, the study aims to determine the strength (q) and temporary support distance (L) required for the hydraulically driven, high-strength temporary support.

A new type of hydraulically driven, high-strength temporary support device has been developed to achieve both high-strength and active temporary support. This system not only meets the requirement of providing active roof protection with a strong initial support force, but it is also specifically designed for soft rock roadways and can effectively control the initial separation.

## Analytical theory

### Establishment of the theoretical model

**Figure 5:**
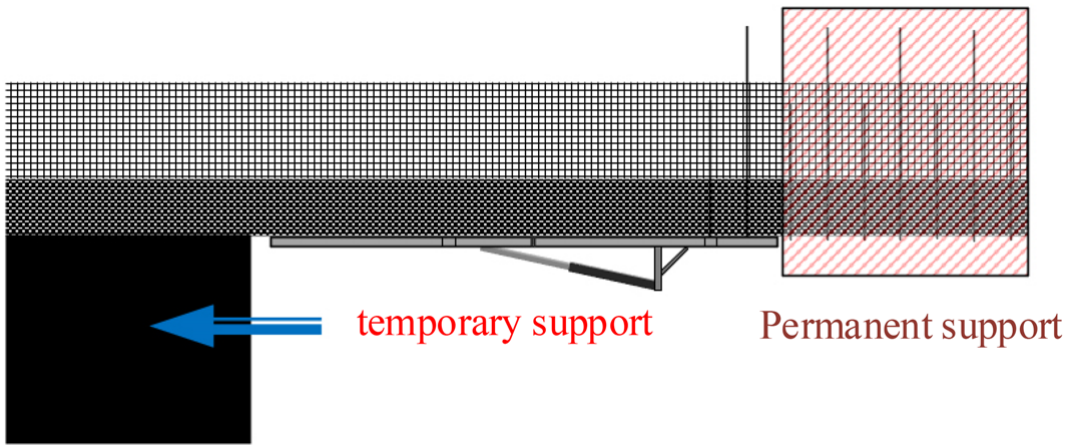
Support characteristics.

Based on the temporary support characteristics during roadway excavation mentioned earlier, the focus of this study was on the top plate of the temporary support section. This is illustrated in Fig. 5.

**Figure 6:**
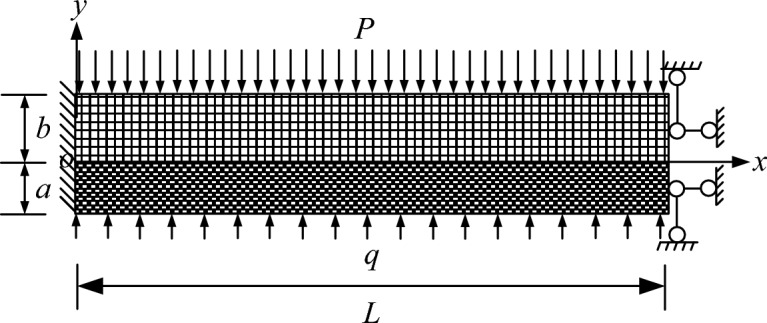
Mechanical model for composite top plate stability analysis.

To establish a mechanical model for the stability analysis of the composite top plate, as shown in Fig. 6, the direct top and old top were selected as the subjects of this study^23–26^. The main focus of this research is to examine the deformation and delamination issues of the top plate. When determining the boundary conditions in Fig. 6, the following factors related to the problem were considered:

The left sideline serves as the boundary restriction for the left boundary, representing the unexcavated position in Fig. 5. It is characterized by solid support, which is its mechanical manifestation.

1 $$ \left\{ \begin{gathered} \left( u \right)_{x = 0,y = 0} = 0 \hfill \\ \left( v \right)_{x = 0,y = 0} = 0 \hfill \\ \left( {\frac{\partial v}{{\partial x}}} \right)_{x = 0,y = 0} = 0 \hfill \\ \end{gathered} \right.. $$

The right limit in Fig. 5 corresponds to the location of the permanent support, which is represented by the right boundary. This boundary is hinged both horizontally and vertically due to the support constraint. As a result, it restricts movement in the x-direction and vertically. The mechanical expression for this boundary is formulated as follows:

2 $$ \left\{ \begin{gathered} \left( u \right)_{x = L,y = 0} = 0 \hfill \\ \left( v \right)_{x = L,y = 0} = 0 \hfill \\ \end{gathered} \right.. $$

Both the upper and lower limits of the model are considered load boundaries. The upper boundary load is determined by the key overlying rock layer, denoted as P = ρgH, where H represents the equivalent overburden height and ρ represents the average density of the overlying rock layer.

**Figure 7:**
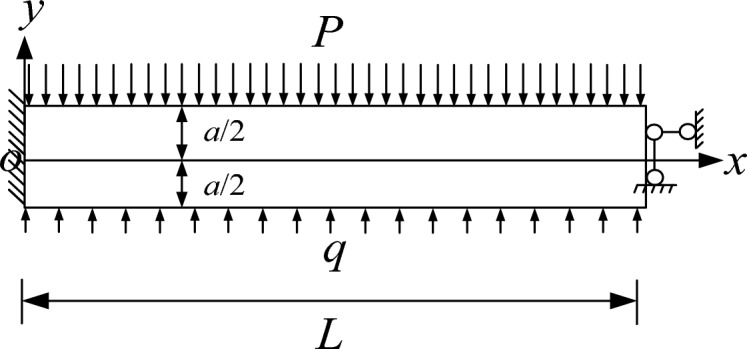
Model for single-layer top slab stability in mechanical analysis.

To address the stability issue of the composite top plate under load in Fig. 6 more effectively, it is crucial to separate the composite top plate model and create a dedicated mechanical analysis model for the stability of the single-layer top plate. This is depicted in Fig. 7. Both the single-layer plate a and single-layer plate b stability fractal mechanical models can be represented by the model in Fig. 7, with the model parameters being the same as those in Fig. 6. It should be noted that when transitioning from the model in Fig. 6 to the model in Fig. 7, the load effect of the upper top plate b.

### Theoretical model resolution

The stress-deformation components of the single-layer slab model in Fig. 7 are solved using a semi-inverse solution. Based on the force characteristics of the top slab model in Fig. 7, it is understood that the stress σx along the x-direction is solely caused by bending moment, the shear stress τxy is solely caused by shear force, and the stress σy along the y-direction is caused by both the overlying rock load P and the temporary support load q. In practical engineering scenarios, P and q are considered constants independent of the x-direction. Since σy is assumed to be a function of y only, the following is considered:

3 $$ \sigma_{y} = f\left( y \right). $$

The stress function φ satisfies the compatibility equation if and only if we have the following:

4 $$ \sigma_{y} = \frac{{\partial^{2} \phi }}{{\partial x^{2} }} = f\left( y \right). $$

By combining both sides of Eq. (4), we obtain:

5 $$ \phi = \frac{{x^{2} }}{2}f\left( y \right) + xf_{1} \left( y \right) + f_{2} \left( y \right). $$

For the stress function given by Eq. (5) to satisfy the compatibility equation, the following conditions must be met:

6 $$ \left\{ \begin{array}{*{20}l} f\left( y \right) = Ay^{3} + By^{2} + Cy + D \hfill \\ f_{1} \left( y \right) = Ey^{3} + Fy^{2} + Gy \hfill \\ f_{2} \left( y \right) = - \frac{A}{10}y^{5} - \frac{B}{6}y^{4} + Jy^{3} + Ky^{2} \hfill \\ \end{array} \right.. $$

The constants A, B, C, D, E, F, G, J, and K.

When substituting Eq. (6) into Eq. (5), the expression for the stress function φ is modified and can be written as follows:

7 $$ \varphi = \frac{{x^{2} }}{2}\left( {Ay^{3} + By^{2} + Cy + D} \right) + x\left( {Ey^{3} + Fy^{2} + Gy} \right) - \frac{A}{10}y^{5} - \frac{B}{6}y^{4} + Jy^{3} - Ky^{2} . $$

The formulation of the stress component can be determined by using Eq. (7) as follows:

8 $$ \left\{ {\begin{array}{*{20}l} {\sigma_{x} { = }\frac{{ \, \partial^{2} \phi }}{{\partial y^{2} }}{ = }\frac{{x^{2} }}{2}(6Ay + 2B) + x(6Ey + 2F) - 2Ay^{3} - 2By^{2} + 6Jy + 2K} \hfill \\ {\sigma_{y} { = }\frac{{ \, \partial^{2} \phi }}{{\partial x^{2} }}{ = }AY^{3} + By^{2} + Cy + D} \hfill \\ {\tau_{{xy^{\prime } }} { = } - \frac{{ \, \partial^{2} \phi }}{\partial xy}{ = } - x\left( {3Ay^{2} + 2By + C} \right) - \left( {3Ey^{2} + 2Fy + G} \right)} \hfill \\ \end{array} } \right.. $$

Based on the upper and lower boundary conditions of the model,

9 $$ \left\{ \begin{array}{*{20}l} \left( {\sigma_{y} } \right)_{{y = \frac{a}{2}}} = - \rho gH \hfill \\ \left( {\sigma_{y} } \right)_{{y = - \frac{a}{2}}} = - q \hfill \\ \left( {\tau_{xy} } \right)_{{y = \pm \frac{a}{2}}} = 0 \hfill \\ \end{array} \right.. $$

To incorporate the upper and lower boundary conditions of the model, Eq. (8) is modified and yields Eq. (9), resulting in:

10 $$ \left\{ \begin{array}{*{20}l} A\frac{{a^{3} }}{8} + B\frac{{a^{2} }}{4} + C\frac{a}{2} + D = - \rho gH \hfill \\ - A\frac{{a^{3} }}{8} + B\frac{{a^{2} }}{4} - C\frac{a}{2} + D = - q \hfill \\ - x\left( {3A\frac{{a^{2} }}{4} + 2B\frac{a}{2} + C} \right) - \left( {3E\frac{{a^{2} }}{4} + 2F\frac{a}{2} + G} \right) = 0 \hfill \\ - x\left( {3A\frac{{a^{2} }}{4} - 2B\frac{a}{2} + C} \right) - \left( {3E\frac{{a^{2} }}{4} - 2F\frac{a}{2} + G} \right) = 0 \hfill \\ \end{array} \right.. $$

The model depicted in Fig. 6, $\left( {\tau_{xy} } \right)_{{y = \pm \frac{a}{2}}}$ is equal to 0 for all values of x, indicating that it is not correlated with

11 $$ \left\{ \begin{array}{*{20}l} E = F = G = B = 0 \hfill \\ 3A\frac{{a^{2} }}{4} + C = 0 \hfill \\ \frac{1}{8}Aa^{3} + C\frac{a}{2} + D = - \rho gH \hfill \\ - \frac{1}{8}Aa^{3} - C\frac{a}{2} + D = - q \hfill \\ C = - \frac{3}{4}Aa^{2} \hfill \\ \end{array} \right.. $$

The solutions to the set of Eq. (11) are as follows:

12 $$ \left\{ \begin{array}{*{20}l} D = - \frac{\rho gH + q}{2} \hfill \\ A = \frac{{2\left( {\rho gH - q} \right)}}{{a^{3} }} \hfill \\ C = - \frac{3}{2}\frac{{\left( {\rho gH - q} \right)}}{a} \hfill \\ B = E = F = G = 0 \hfill \\ \end{array} \right.. $$

By substituting Eq. (12) into Eq. (8), we obtain the following stress components for the model depicted in Fig. 7:

13 $$ \left\{ {\begin{array}{*{20}l} {\sigma_{x} = \frac{6(\rho gH - q)}{{a^{3} }}x^{2} y - \frac{4(\rho gH - q)}{{a^{3} }}y^{3} + 6Jy + 2K} \hfill \\ {\sigma_{y} = \frac{2(\rho gH - q)}{{a^{3} }}y^{3} - \frac{3}{2}\frac{(\rho gH - q)}{a}y - \frac{\rho gH + q}{2}} \hfill \\ {\tau_{xy} = - \frac{6(\rho gH - q)}{{a^{3} }}xy^{2} + \frac{3}{2}\frac{(\rho gH - q)}{a}x} \hfill \\ \end{array} } \right.. $$

The physical and geometric equations used in this analysis are based on the principles of elastic mechanics.

14 $$ \left\{ \begin{array}{*{20}l} {\text{Geometric equation:}}\;\varepsilon_{x} = \frac{\partial u}{{\partial x}},\varepsilon_{y} = \frac{\partial v}{{\partial y}},\gamma_{xy} = \frac{\partial v}{{\partial x}} + \frac{\partial u}{{\partial y}} \hfill \\ {\text{Physical equation:}}\;\varepsilon_{x} = \frac{1}{E}\left( {\sigma_{x} - \mu \sigma_{y} } \right),\varepsilon_{y} = \frac{1}{E}\left( {\sigma_{y} - \mu \sigma_{x} } \right),\gamma_{xy} = \frac{{2\left( {1 + \mu } \right)}}{E}\tau_{xy} \hfill \\ \end{array} \right.. $$

By substituting Eq. (13) into Eq. (14), we obtain:

15 $$ \left\{ \begin{gathered} \varepsilon_{x} { = }\frac{\partial u}{{\partial x}}{ = }\frac{1}{E}\left[ \begin{gathered} \left( {\frac{{6\left( {\rho gH - q} \right)}}{{a^{3} }}x^{2} y - \frac{{4\left( {\rho gH - q} \right)}}{{a^{3} }}y^{3} + 6Hy + 2K} \right) \hfill \\ - \mu \left( {\frac{{2\left( {\rho gH - q} \right)}}{{a^{3} }}y^{3} - \frac{3}{2}\frac{{\left( {\rho gH - q} \right)}}{a}y - \frac{\rho gH + q}{2}} \right) \hfill \\ \end{gathered} \right] \hfill \\ \varepsilon_{y} { = }\frac{\partial v}{{\partial y}}{ = }\frac{1}{E}\left[ \begin{gathered} \left( {\frac{{2\left( {\rho gH - q} \right)}}{{a^{3} }}y^{3} - \frac{3}{2}\frac{{\left( {\rho gH - q} \right)}}{a}y - \frac{\rho gH + q}{2}} \right) \hfill \\ - \mu \left( {\frac{{6\left( {\rho gH - q} \right)}}{{a^{3} }}x^{2} y - \frac{{4\left( {\rho gH - q} \right)}}{{a^{3} }}y^{3} + 6Hy + 2K} \right) \hfill \\ \end{gathered} \right] \hfill \\ \end{gathered} \right.. $$

The two sides of Eq. (15) are integrated to yield:

16 $$ \begin{gathered} u = \frac{1}{E}\left[ \begin{gathered} \left( {\frac{{2\left( {\rho gH - q} \right)}}{{a^{3} }}x^{3} y - \frac{{4\left( {\rho gH - q} \right)}}{{a^{3} }}xy^{3} + 6Hxy + 2Kx} \right) \hfill \\ - \mu \left( {\frac{{2\left( {\rho gH - q} \right)}}{{a^{3} }}xy^{3} - \frac{3}{2}\frac{{\left( {\rho gH - q} \right)}}{a}xy - \frac{\rho gH + q}{2}x} \right) + g_{1} \left( y \right) \hfill \\ \end{gathered} \right] \hfill \\ v = \frac{1}{E}\left[ \begin{gathered} \left( {\frac{{\left( {\rho gH - q} \right)}}{{2a^{3} }}y^{4} - \frac{3}{4}\frac{{\left( {\rho gH - q} \right)}}{a}y^{2} - \frac{\rho gH + q}{2}y} \right) \hfill \\ - \mu \left( {\frac{{3\left( {\rho gH - q} \right)}}{{a^{3} }}x^{2} y^{2} - \frac{{\left( {\rho gH - q} \right)}}{{a^{3} }}y^{4} + 3Hy^{2} + 2Ky + g_{2} \left( x \right)} \right) \hfill \\ \end{gathered} \right]. \hfill \\ \end{gathered} $$

As a result, Eq. (14) yields the result that

17 $$ \begin{aligned} \frac{1}{E} & \left[ { - \mu \left( {\frac{{6\left( {\rho gH - q} \right)}}{{a^{3} }}xy^{2} + g_{2}{\prime} \left( x \right)} \right)} \right] \\ & \;\; + \frac{1}{E}\left[ \begin{gathered} \left( {\frac{{2\left( {\rho gH - q} \right)}}{{a^{3} }}x^{3} - \frac{{12\left( {\rho gH - q} \right)}}{{a^{3} }}xy^{2} + 6Hx} \right) \hfill \\ - \mu \left( {\frac{{6\left( {\rho gH - q} \right)}}{{a^{3} }}xy^{2} - \frac{3}{2}\left( {\frac{\rho gH - q}{a}} \right)x + g_{1}{\prime} \left( y \right)} \right) \hfill \\ \end{gathered} \right] \\ & \; = \frac{{2\left( {1 + \mu } \right)}}{E}\left[ { - \frac{{6\left( {\rho gH - q} \right)}}{{a^{3} }}xy^{2} + \frac{3}{2}\left( {\frac{\rho gH - q}{a}} \right)x} \right]. \\ \end{aligned} $$

The result of simplifying Eqs. (17) is

18 $$ \begin{aligned} \frac{1}{E}g_{2}^{\prime } (x) & + \frac{1}{E}\left[ {\left( {\frac{2(\rho gH - q)}{{a^{3} }}x^{3} + 6Jx} \right) + \frac{3}{2}\mu \left( {\frac{\rho gH - q}{a}} \right)x + g_{1}^{\prime } (y)} \right] \\ = & \frac{(1 + \mu )}{E}\frac{3(\rho gH - q)}{a}x, \\ \end{aligned} $$

i.e.

19 $$ - g_{1}^{\prime } (y) = g_{2}^{\prime } (x) + \frac{2(\rho gH - q)}{{a^{3} }}x^{3} + 6Jx + \frac{3}{2}\mu \left( {\frac{\rho gH - q}{a}} \right)x - 3(1 + \mu )\frac{(\rho gH - q)}{a}x, $$

cause Eq. (19) can result in:

20 $$ - g_{1}^{\prime } (y) = g_{2}^{\prime } (x) + 2Mx^{3} + 6Jx + \frac{3}{2}\mu Ma^{2} x - 3(1 + \mu )Ma^{2} x. $$

Integrate to obtain by setting both sides of Eq. (20) equal to the constant $\omega_{0}$.

21 $$ \left\{ \begin{array}{*{20}l} g_{1} (y) = - \omega_{0} y + C_{1} \hfill \\ g_{2} (x) = - \frac{M}{2}x^{4} + 3Jx^{2} - \left( {\frac{3}{2} + \frac{3}{4}\mu } \right)Ma^{2} x^{2} + \omega_{0} x + C_{2} \hfill \\ \end{array} \right.. $$

While doing so, the boundary condition on the left side of the model Eq. (1) is produced by substituting Eq. (21) into Eq. (16).

22 $$ \left\{ \begin{array}{*{20}l} \left( u \right)_{x = 0,y = 0} = 0 \Rightarrow C_{1} = 0 \hfill \\ \left( v \right)_{x = 0,y = 0} = 0 \Rightarrow C_{2} = 0 \hfill \\ \left( {\frac{\partial v}{{\partial x}}} \right)_{x = 0,y = 0} = 0 \Rightarrow \omega_{0} = 0 \hfill \\ \end{array} \right.. $$

Equation (2) for the right-side boundary condition yields the following result:

23 $$ \left\{ \begin{array}{*{20}l} \left( u \right)_{x = L,y = 0} = 0 \Rightarrow K = - \frac{{\mu \left( {\rho gH + q} \right)}}{4} \hfill \\ \left( v \right)_{x = L,y = 0} = 0 \Rightarrow J = \left( {\frac{1}{2} + \frac{1}{4}\mu } \right)\frac{\rho gH - q}{a} + \frac{{\left( {\rho gH - q} \right)}}{{6a^{3} }}L^{2} \hfill \\ \end{array} \right.. $$

The elastic mechanical solution of the single-story top slab stability mechanical model shown in Fig. 7 can be found using Eqs. (12), (22), and (23).

where the stress factors are located:

24 $$ \left\{ \begin{aligned} \sigma_{x} = & \frac{{6\left( {\rho gH - q} \right)}}{{a^{3} }}x^{2} y - \frac{{4\left( {\rho gH - q} \right)}}{{a^{3} }}y^{3} \\ & + 6\left[ {\left( {\frac{1}{2} + \frac{1}{4}\mu } \right)\frac{\rho gH - q}{a} + \frac{{\left( {\rho gH - q} \right)}}{{6a^{3} }}L^{2} } \right]y - \frac{{\mu \left( {\rho gH + q} \right)}}{2} \\ \sigma_{y} = & \frac{{2\left( {\rho gH - q} \right)}}{{a^{3} }}y^{3} - \frac{3}{2}\frac{\rho gH - q}{a}y - \frac{\rho gH + q}{2} \\ \tau_{xy} = & - \frac{{6\left( {\rho gH - q} \right)}}{{a^{3} }}xy^{2} + \frac{3}{2}\frac{{\left( {\rho gH - q} \right)}}{a}x \\ \end{aligned} \right.. $$

The factor of displacement:

25 $$ \left\{ \begin{gathered} u = \frac{1}{E}\left[ \begin{gathered} \left( \begin{gathered} \frac{{2\left( {\rho gH - q} \right)}}{{a^{3} }}x^{3} y - \frac{{4\left( {\rho gH - q} \right)}}{{a^{3} }}xy^{3} \hfill \\ + 6\left( {\left( {\frac{1}{2} + \frac{1}{4}\mu } \right)\frac{\rho gH - q}{a} + \frac{{\left( {\rho gH - q} \right)}}{{6a^{3} }}L^{2} } \right)xy - \frac{{\mu \left( {\rho gH + q} \right)}}{2}x \hfill \\ \end{gathered} \right) \hfill \\ - \mu \left( {\frac{{2\left( {\rho gH - q} \right)}}{{a^{3} }}y^{3} - \frac{3}{2}\frac{\rho gH - q}{a}y - \frac{\rho gH + q}{2}x} \right) \hfill \\ \end{gathered} \right] \hfill \\ \nu = \frac{1}{E}\left[ \begin{gathered} \left( {\frac{\rho gH - q}{{2a^{3} }}y^{4} - \frac{3}{4}\frac{\rho gH - q}{a}y^{2} - \frac{\rho gH + q}{2}y} \right) - \mu \left( \begin{gathered} \frac{{3\left( {\rho gH - q} \right)}}{{a^{3} }}x^{2} y^{2} \hfill \\ - \frac{{\left( {\rho gH - q} \right)}}{{a^{3} }}y^{4} \hfill \\ \end{gathered} \right) \hfill \\ \left. { + 3\left( {\left( {\frac{1}{2} + \frac{1}{4}\mu } \right)\frac{\rho gH - q}{a} + \frac{{\left( {\rho gH - q} \right)}}{{6a^{3} }}L^{2} } \right)y^{2} - \frac{{\mu \left( {\rho gH + q} \right)}}{2}y} \right) \hfill \\ + \left( { - \frac{\rho gH - q}{{2a^{3} }}} \right. + 3\left( {\left( \frac{1}{2} \right.} \right.\left. { + \frac{1}{4}\mu } \right)\frac{\rho gH - q}{a}\left. { + \frac{{\left( {\rho gH - q} \right)}}{{6a^{3} }}L^{2} } \right)x^{2} \hfill \\ - \left( {\frac{3}{2} + \frac{3}{4}\mu } \right)\frac{\rho gH - q}{a}x^{2} \hfill \\ \end{gathered} \right] \hfill \\ \end{gathered} \right. $$

### Results analysis

#### A mechanical examination of the single-layer top slab model

In the context of the instability of the single-layer top slab, it is primarily seen as structural breakage and material fracture instability. This can be understood through the mechanical model of the single-layer plate, as depicted in Fig. 7. The calculation of the instability problem of the single-layer top slab utilizes the Mohr–Coulomb (M-C) criterion, which is represented by Eq. (26). According to this criterion, damage to the top slab occurs when the ratio of shear stress to normal stress on the shear surface of the top slab reaches its maximum.

26 $$ \tau_{\max } = \sqrt {\left( {\frac{{\sigma_{x} - \sigma_{y} }}{2}} \right)^{2} + \tau_{xy}^{2} } . $$

Equation (24) is substituted into Eq. (26) to produce

27 $$ \begin{aligned} 4\tau_{\max }^{2} = & \left\{ \begin{gathered} \frac{{6\left( {\rho gH - q} \right)}}{{a^{3} }}x^{2} y^{2} - \frac{{6\left( {\rho gH - q} \right)}}{{a^{3} }}y^{3} \hfill \\ + 6\left[ {\left( {\frac{3}{4} + \frac{1}{4}\mu } \right)\frac{\rho gH - q}{a} + \frac{{\left( {\rho gH - q} \right)}}{{6a^{3} }}L^{2} } \right]y \hfill \\ + \frac{{\left( {1 - \mu } \right)}}{2}\left( {\rho gH + q} \right) \hfill \\ \end{gathered} \right\}^{2} \\ & + 4\left[ { - \frac{{6\left( {\rho gH - q} \right)}}{{a^{3} }}xy^{2} + \frac{3}{2}\frac{{\left( {\rho gH - q} \right)}}{a}x} \right]^{2} \\ \end{aligned} $$

From this mechanical model, it becomes apparent that the first occurrence of yield damage will happen at the boundary location when we treat the top plate of the real rock formation as a uniform material without taking fractures into account. We can achieve this by differentiating concerning x and treating y as a constant. To keep things concise, we can represent this as:

28 $$ \left\{ \begin{array}{*{20}l} \left\{ \begin{gathered} \frac{{6\left( {\rho gH - q} \right)}}{{a^{3} }}x^{2} y^{2} - \frac{{6\left( {\rho gH - q} \right)}}{{a^{3} }}y^{3} + 6\left[ {\left( {\frac{3}{4} + \frac{1}{4}\mu } \right)\frac{\rho gH - q}{a} + \frac{{\left( {\rho gH - q} \right)}}{{6a^{3} }}L^{2} } \right]y \hfill \\ + \frac{{\left( {1 - \mu } \right)}}{2}\left( {\rho gH + q} \right) \hfill \\ \end{gathered} \right\} = A \hfill \\ \left[ { - \frac{{6\left( {\rho gH - q} \right)}}{{a^{3} }}xy^{2} + \frac{3}{2}\frac{{\left( {\rho gH - q} \right)}}{a}x} \right] = B \hfill \\ \end{array} \right.. $$

Thus, we can discover:

29 $$ \frac{{\partial \tau_{\max } }}{\partial x} = \frac{{2A\left( {\frac{{12\left( {\rho gH - q} \right)}}{{a^{3} }}2xy^{2} } \right) + 8B\left( { - \frac{{6\left( {\rho gH - q} \right)y^{2} }}{{a^{3} }} + \frac{3}{2}\frac{{\left( {\rho gH - q} \right)}}{a}} \right)}}{{8\tau_{\max } }}. $$

The following conditions must be true where the upper and lower limits were destroyed:

30 $$ \frac{{\partial \tau_{\max } }}{\partial x} = 0. $$

The answer provides:

31 $$ x = \sqrt {\left\{ \begin{array}{*{20}l} \frac{{\left[ { - \frac{{6\left( {\rho gH - q} \right)y^{2} }}{{a^{3} }} + \frac{3}{2}\frac{{\left( {\rho gH - q} \right)}}{a}} \right] \cdot a^{3} }}{{6\left( {\rho gH - q} \right)y^{2} }} \hfill \\ - 6\left[ {\left( { - \frac{3}{4} + \frac{1}{4}\mu } \right)\frac{{\left( {\rho gH - q} \right)}}{a} + \frac{{\left( {\rho gH - q} \right)L^{2} }}{{6a^{3} }}} \right] \times y \hfill \\ - \frac{{\left( {1 - \mu } \right)}}{2}\left( {\rho gH + q} \right) + \frac{{6\left( {\rho gH - q} \right)y^{3} }}{{a^{3} }} \hfill \\ \end{array} \right\} \times \frac{{a^{3} }}{{6\left( {\rho gH - q} \right)y^{2} }}} . $$

The damage location in the x-direction coordinates can be found by putting into Eq. (31) for the upper and lower bounds of the beam.

32 $$ \left\{ \begin{array}{*{20}l} x = \sqrt {\left[ {\left( {2 - \frac{1}{2}\mu + \frac{{L^{2} }}{{9a^{2} }}} \right) - \frac{1 - \mu }{2} \cdot \frac{{\left( {\rho gH + q} \right)}}{{\left( {\rho gH - q} \right)}}} \right] \cdot a} {; }\left( {y = + \frac{a}{2}} \right) \hfill \\ x = \sqrt {\left[ { - \left( {2 - \frac{1}{2}\mu + \frac{{L^{2} }}{{9a^{2} }}} \right) - \frac{1 - \mu }{2} \cdot \frac{{\left( {\rho gH + q} \right)}}{{\left( {\rho gH - q} \right)}}} \right] \cdot a} {; }\left( {y = + \frac{a}{2}} \right) \hfill \\ \end{array} \right.. $$

Assume that the roof rock material’s compressive strength is and its tensile strength is $\sigma_{{\text{t}}}$. In addition, let’s simplify the representation by

33 $$ \left\{ \begin{gathered} \sqrt {\left[ {\left( {2 - \frac{1}{2}\mu + \frac{{L^{2} }}{{9a^{2} }}} \right) - \frac{1 - \mu }{2} \cdot \frac{{\left( {\rho gH + q} \right)}}{{\left( {\rho gH - q} \right)}}} \right] \cdot a} { = }R \hfill \\ \sqrt {\left[ { - \left( {2 - \frac{1}{2}\mu + \frac{{L^{2} }}{{9a^{2} }}} \right) - \frac{1 - \mu }{2} \cdot \frac{{\left( {\rho gH + q} \right)}}{{\left( {\rho gH - q} \right)}}} \right] \cdot a} { = }T \hfill \\ \end{gathered} \right.. $$

The expression of the maximum primary stress in the top plate is obtained by the mechanics of materials as follows:

34 $$ \begin{array}{*{20}c} {\sigma_{1} } \\ {\sigma_{2} } \\ \end{array} = \frac{{\sigma_{x} + \sigma_{y} }}{2} \pm \left( {\sqrt {\left( {\frac{{\sigma_{x} - \sigma_{y} }}{2}} \right)^{2} + \tau_{xy}^{2} } } \right), $$

where denotes the top plate’s maximum tensile stress, which is placed on its lower surface at $y = - \frac{a}{2}$, and denotes the top plate’s maximum compressive stress, which is located on its lower surface at $y = \frac{a}{2}$.

The top slab’s breaking condition is determined for the brittle rock material by applying the first strength theory, i.e.

35 $$ \left\{ \begin{gathered} \sigma_{1} { = }\sigma_{{\text{t}}} \hfill \\ \sigma_{2} { = }\sigma_{{\text{c}}} \hfill \\ \end{gathered} \right.. $$

The fracture of the single-layer top slab can be determined by substituting Eq. (24) and the values of the damage points x and y (32) into Eqs. (34) and (35). This will provide the crucial conditions for fracture.

36 $$ \frac{{\sqrt {\begin{array}{*{20}l} {\left\{ \begin{array}{*{20}l} \frac{{6\left( {\rho gH - q} \right)}}{{a^{3} }}T^{2} \left( { - \frac{a}{2}} \right)^{2} - \frac{{6\left( {\rho gH - q} \right)}}{{a^{3} }}\left( { - \frac{a}{2}} \right)^{3} \hfill \\ + 6\left[ {\left( {\frac{3}{4} + \frac{1}{4}\mu } \right)\frac{\rho gH - q}{a} + \frac{{\left( {\rho gH - q} \right)}}{{6a^{3} }}L^{2} } \right]\left( { - \frac{a}{2}} \right) + \frac{{\left( {1 - \mu } \right)}}{2}\left( {\rho gH + q} \right) \hfill \\ \end{array} \right\}} \hfill \\ { + 4\left[ { - \frac{{6\left( {\rho gH - q} \right)}}{{a^{3} }}T\left( { - \frac{a}{2}} \right)^{2} + \frac{3}{2}\frac{{\left( {\rho gH - q} \right)}}{a}T} \right]^{2} } \hfill \\ \end{array} } }}{2} = \sigma_{{\text{t}}} , $$

37 $$ \frac{{\sqrt {\begin{array}{*{20}l} {\left\{ \begin{gathered} \frac{{6\left( {\rho gH - q} \right)}}{{a^{3} }}R^{2} \left( \frac{a}{2} \right)^{2} - \frac{{6\left( {\rho gH - q} \right)}}{{a^{3} }}\left( \frac{a}{2} \right)^{3} \hfill \\ + 6\left[ {\left( {\frac{3}{4} + \frac{1}{4}\mu } \right)\frac{\rho gH - q}{a} + \frac{{\left( {\rho gH - q} \right)}}{{6a^{3} }}L^{2} } \right]\left( \frac{a}{2} \right) + \frac{{\left( {1 - \mu } \right)}}{2}\left( {\rho gH + q} \right) \hfill \\ \end{gathered} \right\}} \\ { + 4\left[ { - \frac{{6\left( {\rho gH - q} \right)}}{{a^{3} }}R\left( \frac{a}{2} \right)^{2} + \frac{3}{2}\frac{{\left( {\rho gH - q} \right)}}{a}R} \right]^{2} } \\ \end{array} } }}{2}{ = }\sigma_{{\text{c}}} . $$

The Eq. (36) can be used to determine the critical condition for the failure of a single-layer roof plate, which is made of a compressive material. This equation represents the important circumstance where the single-layer top slab breaks. The rock mechanical property parameters for the roof plate of the mining face were established through field research and physical and mechanical property tests, as detailed in Table 1.

**Table 1:** Rock mechanical properties of the roof of the excavation workforce.

ρ (kg·m^-3^)	g (N·kg^-1^)	μ	H (m)	σ_t_ (MPa)	σ_c_ (MPa)	a (m)	b (m)
2450	9.8	0.15	10	3.0	20	1.5	6.15

By substituting the data from Table 1 into Eq. (36), the relationship between the support load q and the critical breaking length L of the top plate can be determined as follows:

$$ 317,169.1358L^{2} + (6.012962963 - 0.12345679L^{2} )q = 22,539,577. $$

**Figure 8:**
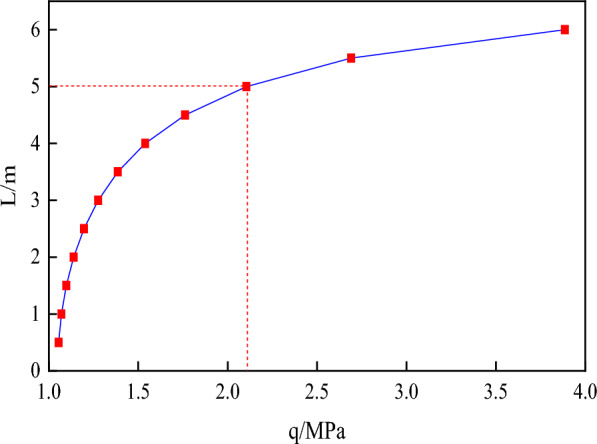
The variation law of temporary support length with temporary support load.

As depicted in Fig. 8, which showcases the outcome of the mentioned equation, it is apparent that in the model of a single-layer roof, there is a notable requirement for a substantial temporary support load to prevent roof failure when the length of temporary support is extremely small. For instance, when the temporary support length L is 0.5 m, a temporary support strength exceeding 1 MPa is imperative. This effectively demonstrates the correlation between the load at which the roof breaks and the length of temporary support in the single-layer roof model, thus emphasizing the significance of temporary support in maintaining roof stability.

#### Building and analyzing a stacked-top plate mechanical analysis model

The mechanical analysis model for stacked-top slabs is derived from the previous mechanical analysis model for single-layer top slabs. In real-life engineering scenarios, the rock layer often breaks and falls apart before experiencing significant elastic–plastic deformation. This happens because the top slab experiences high levels of stress and the rock material is brittle. Therefore, in the mechanical analysis model for laminated top slabs, we don’t consider the separation between slabs as a result of elastic slab warping. Instead, we consider it a result of material fracture near the interface between slabs, where the stress level exceeds the material’s capacity to bear the load.

The occurrence of delamination is predicted when the stress near the interface between the plates exceeds the material’s ability to bear the load, leading to failure in the analysis. This prediction is made based on the assumption that there is complete contact between the interfaces of the upper and lower plates. A mechanical solution is then calculated for the laminated top plate, considering it to be elastic and subjected to a uniform load, with one end fixed and the other end hinged.

38 $$\begin{array}{*{20}l}    \begin{gathered}   \sigma _{x}  = \frac{{6(\rho gH - q)}}{{\left( {a + b} \right)^{3} }}x^{2} \left( {y - \frac{{b - a}}{2}} \right) - \frac{{4(\rho gH - q)}}{{\left( {a + b} \right)^{3} }}\left( {y - \frac{{b - a}}{2}} \right)^{3}  \hfill \\    + 6\left[ {\left( {\frac{1}{2} + \frac{1}{4}\mu } \right)\frac{{\rho gH - q}}{{\left( {a + b} \right)}} + \frac{{(\rho gH - q)}}{{6\left( {a + b} \right)^{3} }}L^{2} } \right]\left( {y - \frac{{b - a}}{2}} \right) - \frac{{\mu (\rho gH + q)}}{2} \hfill \\  \end{gathered}  \hfill  \\    {\sigma _{y}  = \frac{{2(\rho gH - q)}}{{\left( {a + b} \right)^{3} }}\left( {y - \frac{{b - a}}{2}} \right)^{3}  - \frac{3}{2}\frac{{\rho gH - q}}{{\left( {a + b} \right)}}\left( {y - \frac{{b - a}}{2}} \right) - \frac{{\rho gH + q}}{2}} \hfill  \\    \begin{gathered}   \tau _{{xy}}  =  - \frac{{6(\rho gH - q)}}{{\left( {a + b} \right)^{3} }}x\left( {y - \frac{{b - a}}{2}} \right)^{2}  + \frac{3}{2}\frac{{(\rho gH - q)}}{{\left( {a + b} \right)}}x \hfill \\   u = \frac{1}{E}\left[ {\left( {\frac{{2(\rho gH - q)}}{{\left( {a + b} \right)^{3} }}x^{3} \left( {y - \frac{{b - a}}{2}} \right) - \frac{{4(\rho gH - q)}}{{\left( {a + b} \right)^{3} }}x\left( {y - \frac{{b - a}}{2}} \right)^{3} } \right.} \right. \hfill \\   \left. { + 6\left( {\left( {\frac{1}{2} + \frac{1}{4}\mu } \right)\frac{{\rho gH - q}}{{\left( {a + b} \right)}} + \frac{{(\rho gH - q)}}{{6\left( {a + b} \right)^{3} }}L^{2} } \right)x\left( {y - \frac{{b - a}}{2}} \right) - \frac{{\mu (\rho gH + q)}}{2}x} \right) \hfill \\   \left. { - \mu \left( {\frac{{2(\rho gH - q)}}{{\left( {a + b} \right)^{3} }}\left( {y - \frac{{b - a}}{2}} \right)^{3}  - \frac{3}{2}\frac{{\rho gH - q}}{{\left( {a + b} \right)}}\left( {y - \frac{{b - a}}{2}} \right) - \frac{{\rho gH + q}}{2}x} \right)} \right] \hfill \\  \end{gathered}  \hfill  \\    {v = \frac{1}{E}\left[ {\left( {\frac{{\rho gH - q}}{{2\left( {a + b} \right)^{3} }}\left( {y - \frac{{b - a}}{2}} \right)^{4}  - \frac{3}{4}\frac{{\rho gH - q}}{{\left( {a + b} \right)}}\left( {y - \frac{{b - a}}{2}} \right)^{2}  - \frac{{\rho gH + q}}{2}\left( {y - \frac{{b - a}}{2}} \right)} \right)} \right.} \hfill  \\    \begin{gathered}    - \mu \left( {\frac{{3(\rho gH - q)}}{{\left( {a + b} \right)^{3} }}x^{2} \left( {y - \frac{{b - a}}{2}} \right)^{2}  - \frac{{(\rho gH - q)}}{{\left( {a + b} \right)^{3} }}\left( {y - \frac{{b - a}}{2}} \right)^{4} } \right) \hfill \\   \left. { + 3\left( {\left( {\frac{1}{2} + \frac{1}{4}\mu } \right)\frac{{\rho gH - q}}{{\left( {a + b} \right)}} + \frac{{(\rho gH - q)}}{{6\left( {a + b} \right)^{3} }}L^{2} } \right)\left( {y - \frac{{b - a}}{2}} \right)^{2}  - \frac{{\mu (\rho gH + q)}}{2}\left( {y - \frac{{b - a}}{2}} \right)} \right) \hfill \\   \left. { + 3\left( {\left( {\frac{1}{2} + \frac{1}{4}\mu } \right)\frac{{\rho gH - q}}{{\left( {a + b} \right)}} + \frac{{(\rho gH - q)}}{{6\left( {a + b} \right)^{3} }}L^{2} } \right)\left( {y - \frac{{b - a}}{2}} \right)^{2}  - \frac{{\mu (\rho gH + q)}}{2}\left( {y - \frac{{b - a}}{2}} \right)} \right) \hfill \\   \left. {\left. { + \frac{{(\rho gH - q)}}{{6\left( {a + b} \right)^{3} }}L^{2} } \right)x^{2}  - \left( {\frac{3}{2} + \frac{3}{4}\mu } \right)\frac{{\rho gH - q}}{{\left( {a + b} \right)}}x^{2} } \right] \hfill \\  \end{gathered}\end{array}$$

The maximal shear stress theory states.

39 $$ \tau_{\max } = \sqrt {\left( {\frac{{\sigma_{x} - \sigma_{y} }}{2}} \right)^{2} + \tau_{xy}^{2} } $$

Obtained:

40 $$ \begin{aligned} 4\tau_{{_{\max } }}^{2} = & \left\{ \begin{gathered} \frac{6(\rho gH - q)}{{\left( {a + b} \right)^{3} }}x^{2} \left( {y - \frac{b - a}{2}} \right)^{2} - \frac{6(\rho gH - q)}{{\left( {a + b} \right)^{3} }}\left( {y - \frac{b - a}{2}} \right)^{3} \hfill \\ + 6\left[ {\left( {\frac{3}{4} + \frac{1}{4}\mu } \right)\frac{\rho gH - q}{{\left( {a + b} \right)}} + \frac{(\rho gH - q)}{{6\left( {a + b} \right)^{3} }}L^{2} } \right]\left( {y - \frac{b - a}{2}} \right) + \frac{(1 - \mu )}{2}(\rho gH + q) \hfill \\ \end{gathered} \right\}^{2} \\ & + 4\left[ { - \frac{6(\rho gH - q)}{{\left( {a + b} \right)^{3} }}x\left( {y - \frac{b - a}{2}} \right)^{2} + \frac{3}{2}\frac{(\rho gH - q)}{{\left( {a + b} \right)}}x} \right]^{2} . \\ \end{aligned} $$

There are cracks $y = \frac{b - a}{2}$, which are where the top and lower plates meet.

41 $$ 4\tau_{{_{\max } }}^{2} = \left[ {\frac{(1 - \mu )}{2}(\rho gH + q)} \right]^{2} { + }4\left[ {\frac{3}{2}\frac{(\rho gH - q)}{{\left( {a + b} \right)}}x} \right]^{2} . $$

Let represent the material’s shear damage strength. Thus, it is necessary that

42 $$ \sqrt {\left[ {\frac{(1 - \mu )}{2}(\rho gH + q)} \right]^{2} { + }4\left[ {\frac{3}{2}\frac{(\rho gH - q)}{{\left( {a + b} \right)}}L} \right]^{2} } \le 2\sigma_{n3} . $$

I have successfully identified the key factors that contribute to the development of delamination in the stacked top plate. To evaluate this, I have incorporated specific variables related to the site, such as the density (ρ) of 2450 kg/m^3^, gravitational acceleration (g) of 9.8 N/kg, the thickness of the underlying rock layer 1 (a) as 1.5 m, the thickness of the overlying rock layer 2 (b) as 6.15 m, Poisson’s ratio (μ) as 0.15, and the conversion factor (H) as 10 m. In addition, the variables “q” vary from 0 to 20 kN/m2, “L” varies from 0.5 to 3 m, and “σn3” varies from 1 to 3 MPa.

(1) Top plate delamination and the temporary support load q

43 $$ Q = \sqrt {\left[ {\frac{(1 - \mu )}{2}(\rho gH + q)} \right]^{2} { + }4\left[ {\frac{3}{2}\frac{(\rho gH - q)}{{\left( {a + b} \right)}}L} \right]^{2} } . $$

**Figure 9:**
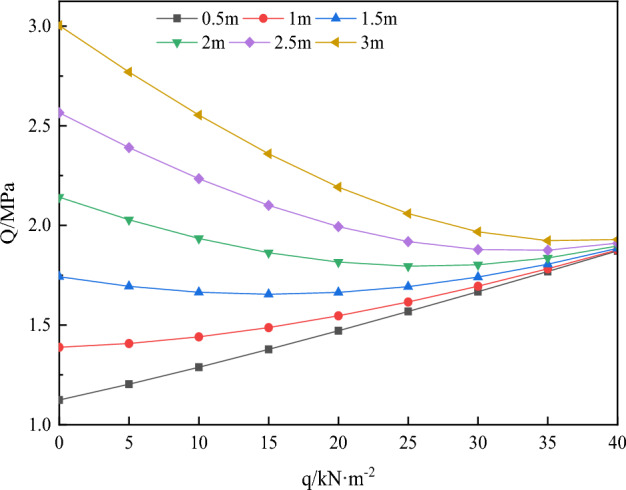
Variation of parameter Q with temporary support strength q.

The formula mentioned earlier is used to determine the change in the parameter Q based on the support load q. The support length L and conditions remain the same as shown in Fig. 9. The parameter Q is an indicator of the difficulty in controlling delamination through support behavior. A higher value of Q suggests that controlling top plate delamination is less favorable.

Figure 9 demonstrates that when there is only one temporary support, the strength of the support increases at distances of 0.5 and 1 m. The parameter Q also increases, although its value remains low. This implies that the condition of the rock surrounding the roof is relatively favorable at this point, indicating that the force exerted by the temporary support has minimal effect on controlling the rock surrounding the roof.

However, when the distance between temporary supports is set at 1.5, 2, 2.5, or 3 m, increasing the strength of the temporary supports from 0 to 30 kN/m^2^ leads to a gradual decrease in the parameter Q. This suggests that the force exerted by the temporary supports has a significant impact on controlling the surrounding rock of the roof during this stage and effectively prevents the initial detachment of the top layer. It is worth noting that a temporary support strength of 10 kN/m^2^ has a stronger influence on the roof when the temporary support distances are 1.5 m and 2 m, but this effect becomes less pronounced as the temporary support strength increases.

(2) The top plate’s departure and the temporary support’s length L.

**Figure 10:**
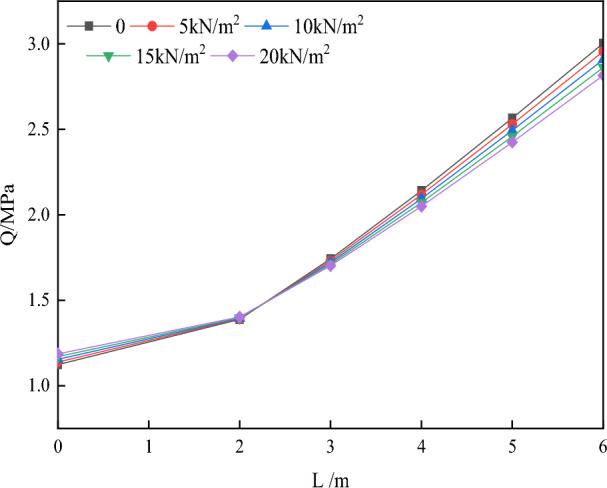
Variation of parameter Q with temporary support length L.

Figure 10 depicts the curve that shows how the parameter Q changes as the support length L varies while keeping the support load q constant. The parameter Q, which is greatly affected by the temporary support length L, suggests that reducing the span of the excavation can significantly improve the initial delamination of the roof of the roadway.

Figure 10 illustrates that when the support length L exceeds 2.2 m, the parameter Q increases at a faster pace with the same temporary support load. This suggests that it is more advantageous to limit the length of a single excavation to within 2.2 m. Furthermore, extending the excavation distance beyond this threshold will lead to a significant rise in both the cost and strength needed for temporary support.

**Figure 11:**
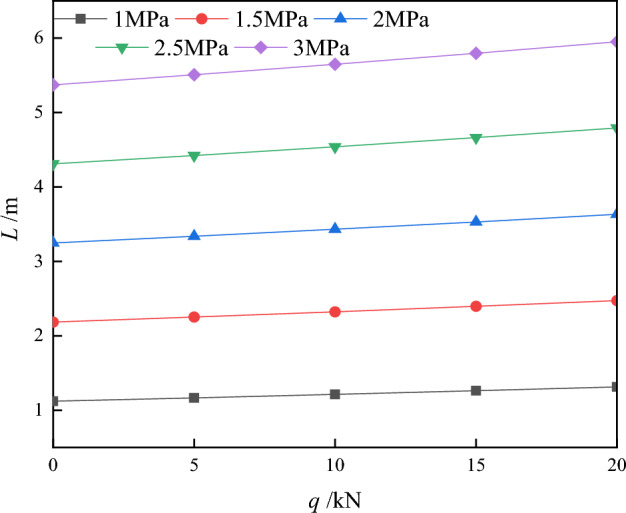
Variation of support strength q and support length L for different shear strengths.

(3) The connection between the load on the support (q) and the length of the support (L) is examined under different shear strengths in the critical condition where delamination happens. Additionally, the relationship between q and L is explored when the following equation is satisfied, as depicted in Fig. 11.

44 $$ Q = \sqrt {\left[ {\frac{(1 - \mu )}{2}(\rho gH + q)} \right]^{2} { + }4\left[ {\frac{3}{2}\frac{(\rho gH - q)}{{\left( {a + b} \right)}}L} \right]^{2} } = 2\sigma_{n3} . $$

According to Fig. 11, it is apparent that increasing the strength of the temporary support can allow for a longer length of temporary support under ideal conditions. This means that the excavation can progress further, and the roof slab surrounding the rock will have a higher shear strength with a longer temporary support length. Moreover, the stability of the top plate is more influenced by the nature of the top plate itself than the strength of the temporary support.

Referring to Figs. 9 and 10, it is recommended to maintain the temporary support strength within the range of 0 to 20 kN/m^2^ for the surrounding rock conditions of the A4027 return airway roof. Additionally, the suggested distance for the temporary support is between 0.8 and 2.2 m.

## The roadway excavation face’s roof stress and deformation in a numerical simulation

### Force analysis and strength test of support beam

#### Support beam force analysis

The active support force of the temporary support is obtained through the lateral installation of the jack. The thrust generated by the jack is calculated by multiplying the pressure resulting from the internal cylinder pressurization by the area of the cylinder. The calculation formula is shown below as (45).

45 $$ F = \pi r^{2} p, $$

where F represents the thrust that the jack can produce, r represents the radius of the internal cylinder of the jack, and p represents the pressure of the internal cylinder. Taking r = 0.0025 m and p = 28 × 10^6^ Pa as the values, substituting the given values into the formula, and calculating the result, the lateral thrust provided by the jack is 54.95 kN.

$$ \left[ {\left( \frac{50}{2} \right) \times 10^{ - 3} } \right]^{2} \pi \times 28 \times 10^{6} = 54950\,{\text{N}}. $$

**Figure 12:**
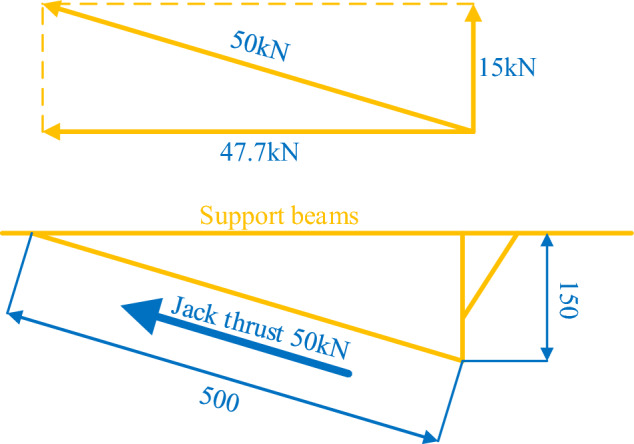
Schematic diagram of the force acting on the support beam by the jack.

The support beam is the main material of the entire temporary support device and is made of No. 10 channel steel. It is simplified as a mechanical structure in the diagram below for force analysis. When the jack is under pressure, the thrust it produces is the product of the cylinder’s cross-sectional area and the pressure. Here, a cylinder with a diameter of 50 mm is selected, and the maximum thrust that the jack can provide is calculated as 50 kN (considering a pressure of 28 MPa, the thrust is calculated as 54.95 kN, approximately 5.5 tons). The thrust of the jack is decomposed, as shown in Fig. 12. Based on the dimensional relationship of the mechanical structure, the support beam is subjected to a vertical force F_y_ from the jack, which can be calculated using formula 4-2 to provide support to the roof.

46 $$ F_{y} = F\cos \theta , $$

where cos θ = 150/500 and F is taken as 50 kN, by substituting these values into formula (46), we can calculate the vertical force Fy as 15 kN. This means that each beam of the active support system for roof protection with high initial bracing can provide an initial support force greater than 15 kN.

**Figure 13:**
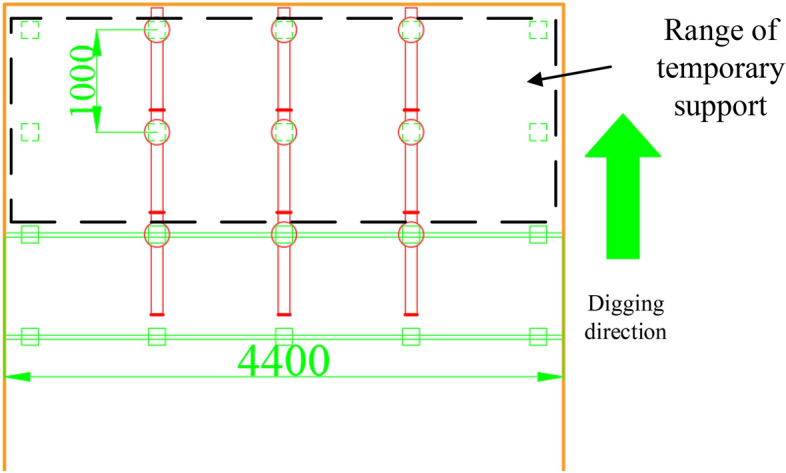
Schematic diagram of the scope of temporary support.

The cross-section of the A4027 return airway is 4.4 m wide. The temporary support range is shown in Fig. 13, with three sets of temporary supports installed. Each set consists of two support beams, making a total of six support beams. Assuming a design advance distance of 1000 mm and a two-mining-one-support method, the roof support area for the active temporary support is shown as a rectangular roof in Fig. 13, measuring 4400 × 2000 mm. Therefore, the calculation formula for the strength q of the active temporary support is shown as (47).

47 $$ q = \frac{{F_{y} }}{S}, $$

where S represents the area of the rectangular surface, which is 4400 × 2000 mm, and Fy is 15 kN, we can substitute these values into the formula for calculation.

$$ \frac{15 \times 6}{{4.4 \times 2}} = 10.2\;{\text{kN}}/{\text{m}}^{2} . $$

The calculated temporary support strength is q = 10.2 kN/m^2^. In the later stage, the selection of the jack model should be greater than the required support strength based on actual conditions. The stiffness of the selected jack is 20 kN/m, which meets the strength requirements.

#### Testing the strength of the support beams

In the application of the complete temporary support device, the support beam fixed at the end of the anchor rod acts as a fixed end, while the support beam extending forward to the empty roof area functions as a cantilever beam. This is by the working principle and structural design of the hydraulically driven, high-strength temporary support device. According to the theory of material mechanics, the end of the cantilever beam, which is under the highest pressure, is the most unstable when the active temporary roof protection device with a strong initial bracing force is near the roof plate.

Therefore, during the strength testing of the support beam, it is sufficient for the entire support beam to pass if the position of the support beam's end near the digging head successfully meets the testing criteria.

**Figure 14:**
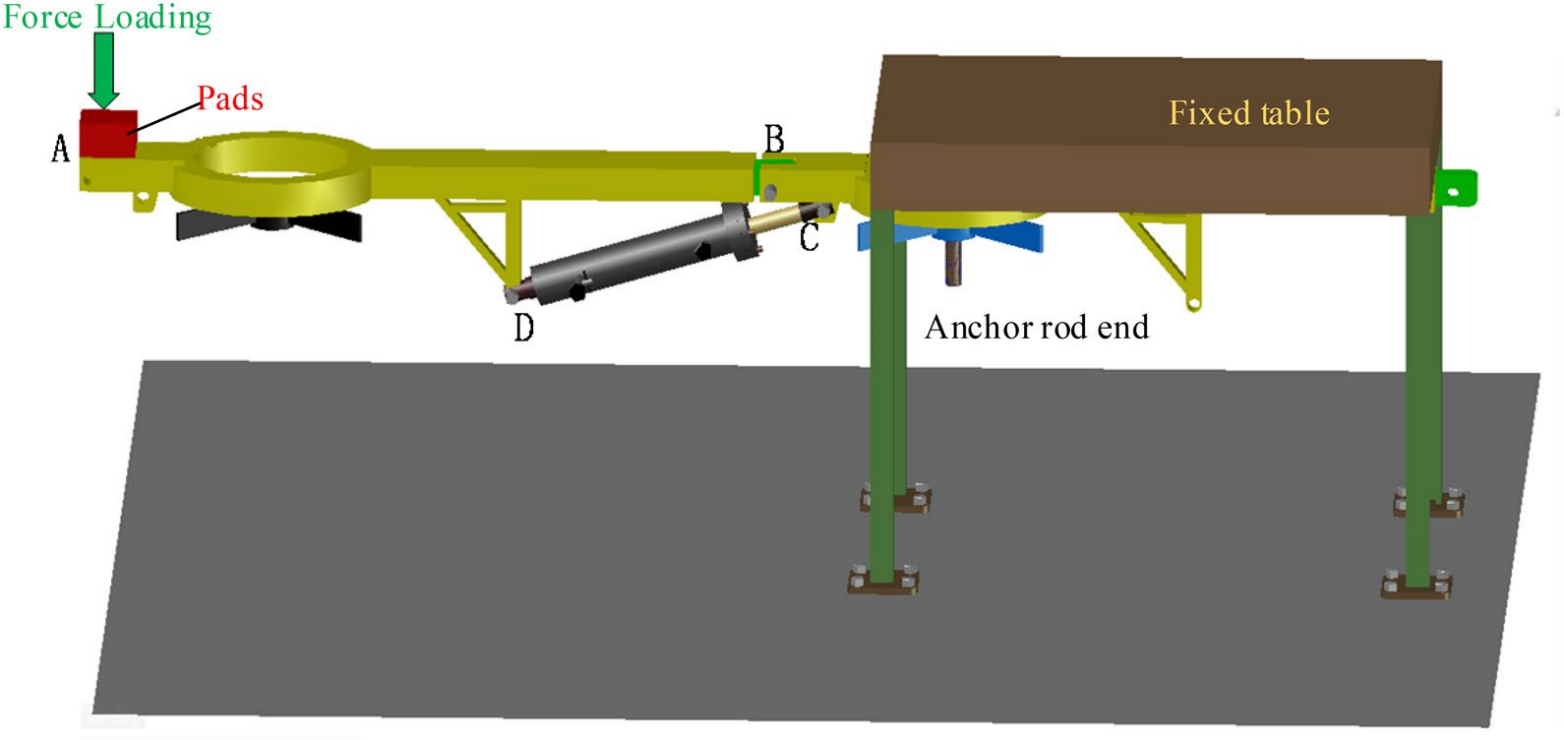
Depicts the strength test procedure.

**Figure 15:**
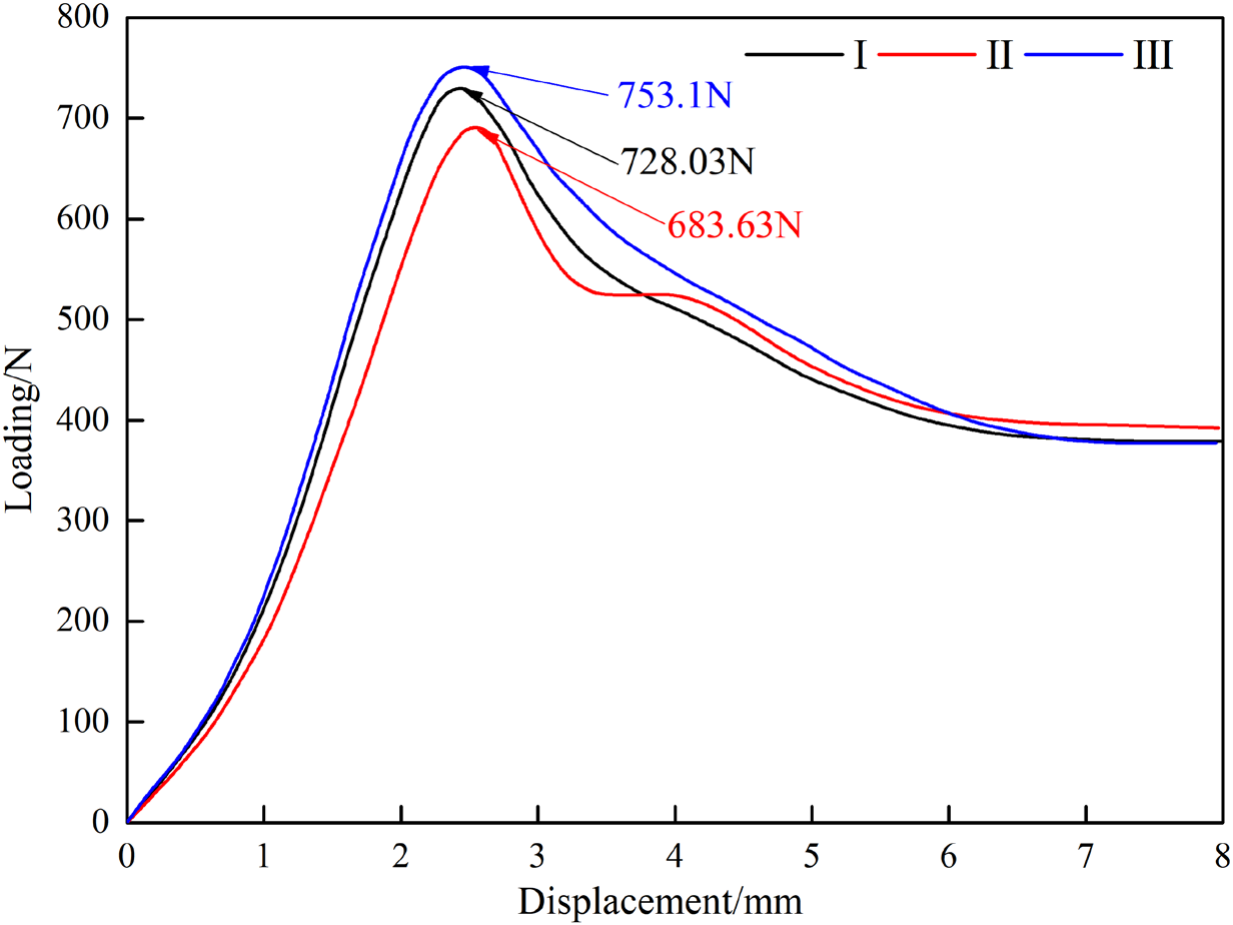
Depicts the experimental data curve for the press.

Figure 14 illustrates the strength test procedure; Table 2 presents the maximum peak load recorded during the experiment; and Fig. 15 displays the experimental data curve for the press.

**Table 2:** The greatest peak load.

Number	Peak load/N	Deformation amount/mm	Destruction point
I	728.03	2.41	C
II	683.63	2.7	C
III	753.1	2.45	C

Analysis of Fig. 15 indicates that the support beam undergoes elastic deformation rather than plastic deformation or weld cracking. However, due to the peak load, the C point of the jack has experienced contraction. Upon dismantling the jack for inspection after the conclusion of the experiment, it was discovered that damage to the jack's hydraulic lock had resulted in the unloading of the jack.

Within the elastic deformation range of the support beam, the pressure strengths at support beam A are 13.01 kN/m^2^, 12.19 kN/m^2^, and 13.45 kN/m^2^. The strength difference between the first and second groups is 10.3%. The compressive strengths that the weakest part of the support beam can withstand are all greater than 12 kN/m^2^. This demonstrates that the entire support beam can withstand the pressure, with the maximum tolerable strength being greater than 12 kN/m^2^.

### Numerical modeling

The A4027 return airway, which has a rectangular section measuring 4.4 m wide and 2.8 m high, is selected as the simulated roadway, taking into account the engineering and geological conditions of the XCMG Xinjiang Saier Six Mine.

In terms of the stress boundary, for the sake of computational efficiency, the model is constrained within a specific range for the top and bottom rock layers, with the upper limit set at Z = 50 m.

Regarding the displacement boundary, normal displacement is applied to the constrained boundary at specific coordinates: X = 0 m, X = 50 m, Y = 0 m, Y = 30 m, and Z = 0 m.

To replace the stress boundary, an equivalent load of 4.98 MPa is applied.

**Figure 16:**
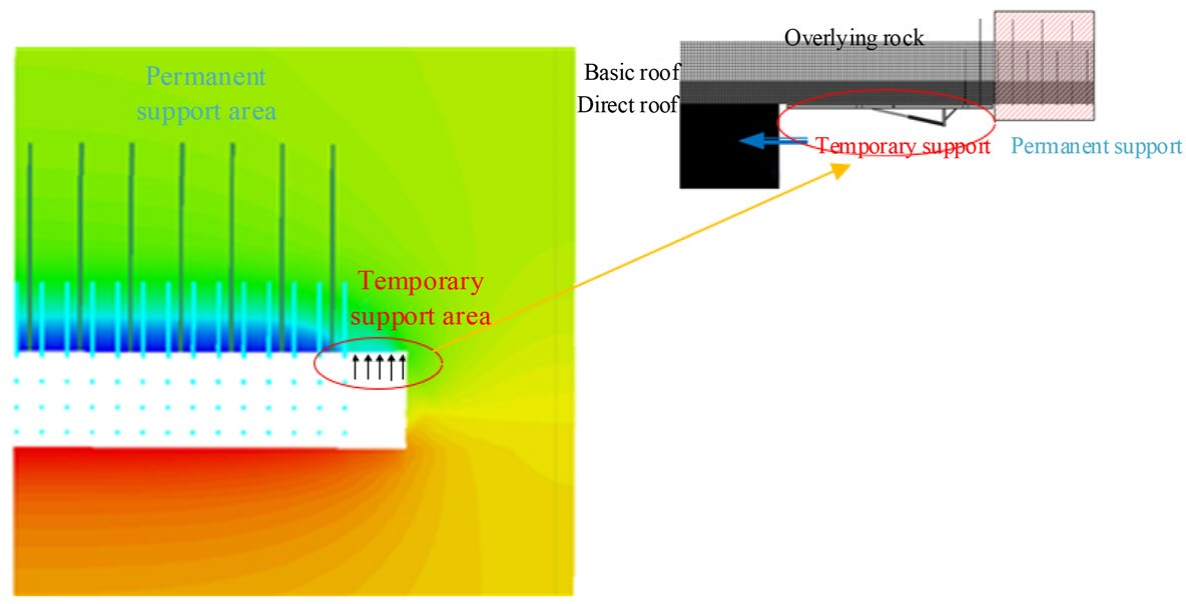
Simplified numerical model.

As depicted in Fig. 16, the 3D numerical model is established with dimensions of L × W × H = 50 m × 50 m × 30 m, taking into account the simplification of the geological conditions at the site. In the numerical simulation, the Mohrs coulomb yield failure criterion is selected as the material damage criterion.

In the simulation of temporary support, the model of the support device is simplified, and the strength of the temporary support device on the roadway’s roof is replaced by a uniform load in the temporary support area of the roadway model. To replicate the exerted supporting force of the active temporary support at the temporary support interface, a uniformly distributed load, represented as q, should be applied. This is intended to mimic the upward supporting force generated by the active temporary support. This allows for the investigation of how the roadway roof will move and how stress will be distributed when varying the temporary support strength (Q) and length (L). The mechanical parameters of the coal rock utilized in the simulation are presented in Table 3.

**Table 3:** Coal rock mechanical parameters.

Lithology	σ_bc_/MPa	σ_t_/MPa	φ/□	c/MPa	υ	ρ (kg/m^3^)
Carbonaceous mudstone	33.3	3.3	36	6.2	0.21	2415
Siltstone	65.4	5.6	45	12.2	0.26	2667
A6 coal	21.1	1.2	31	4.7	0.13	1380
Sandy mudstone	28.0	2.7	25	7.0	0.28	2500
A5 Coal	21.1	1.2	31	4.7	0.13	1380
A4 Coal	21.1	1.2	31	4.7	0.13	1380

### Analysis of the outcomes of numerical simulation

#### The legislation regarding roadway roof deformation length and the strengths of various temporary supports

**Figure 17:**
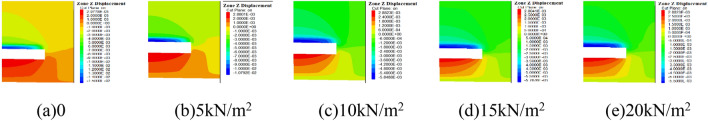
A plot of the roadway roof's vertical displacement clouds.

**Figure 18:**
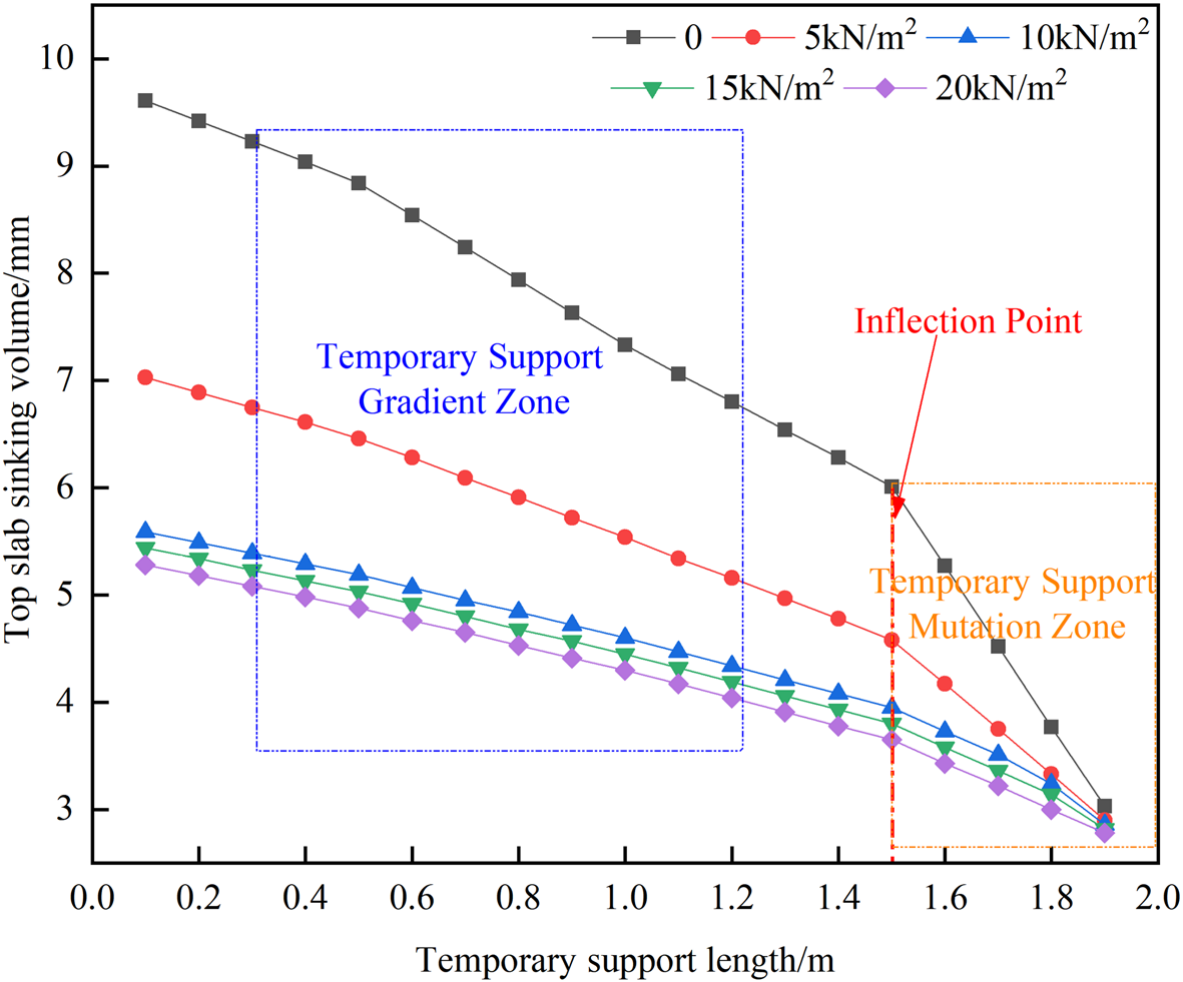
Top slab subsidence curve in the area of temporary support.

Upon analyzing Figs. 17 and 18, it is evident that increasing the active support force leads to a gradual decrease in the maximum sinkage of the top plate, assuming a fixed length of temporary support. When the applied load (Q) is less than 10 kN/m^2^, the maximum sinkage of the top plate decreases significantly from 9.61 to 5.59 mm. This reduction is quite noticeable. Conversely, when Q exceeds 10 kN/m^2^, the sinkage of the top plate is minimal, indicating the effectiveness of the implemented measures to suppress sinking.

By considering the strength of the temporary support, it can be observed that as the length of the temporary support increases, the amount of sinking in the roof gradually decreases. The point of inflection occurs at a length of 1.5 m, where different values of Q exhibit varying effectiveness in preventing roof sinking, resulting in a zone of slow deformation. However, when the length (L) exceeds 1.5 m, the impact of different Q values on the roof sinking becomes less pronounced, leading to a sudden deformation zone. Therefore, it is advisable to select a temporary support length of 2.0 m.

#### The stress distribution characteristics in the roadway’s ceiling vary with different strengths and lengths of temporary support

**Figure 19:**
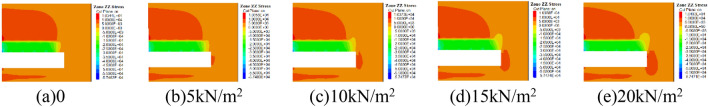
Cloud of vertical tension on the roadway’s roof.

**Figure 20:**
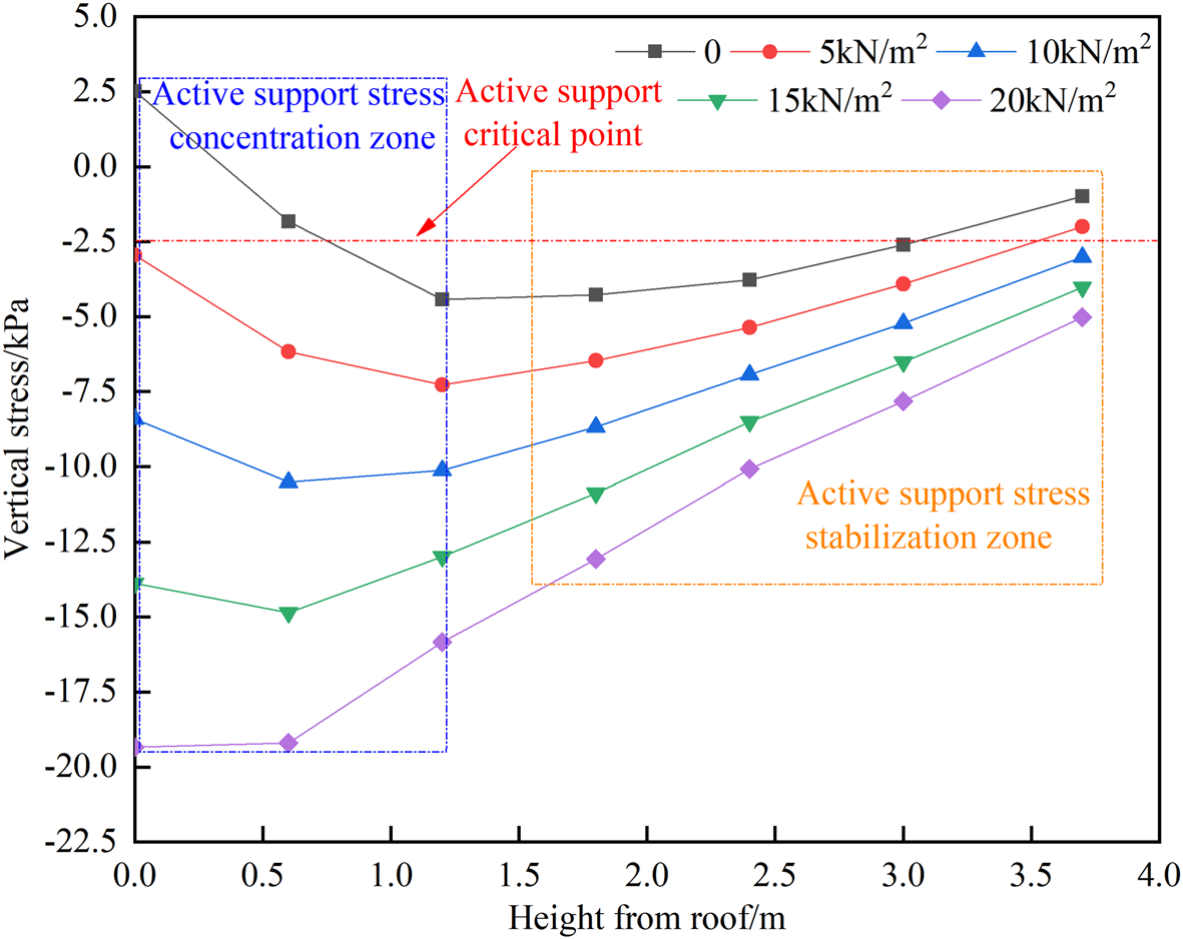
The top plate’s vertical stress curve in the temporary support area.

Upon analyzing Figs. 19 and 20, it was observed that when the length of the temporary support L is provided and the active support force is zero, the first monitoring point on the roadway roof experiences a positive stress of 2.51 kPa, indicating tensile stress in the roof. However, when the active support force is 10 kN/m^2^, the stress in the roadway roof becomes completely negative, indicating compressive stress during the measurement. This demonstrates the impact on stress distribution.

When the strength of the temporary support is known, the vertical stress in the roadway roof initially increases with depth before decreasing. For roof depths less than 1.2 m, different Q values result in a rapid increase in vertical stress, leading to the formation of a stress concentration zone in the temporary support area. However, for depths greater than 1.2 m, the vertical stress decreases smoothly with different Q values, creating a stress stability zone in the temporary support area.

From my perspective, it is clear that the stress distribution can be effectively managed by having a temporary support strength of more than 5 kN/m^2^ in the area where the roadway roof is being supported temporarily. Through a comprehensive estimation method and previous theoretical studies, we have determined that the optimal temporary support strength is 10 kN/m^2^ and the optimal length is 2 m. By using a hydraulic-driven, high-strength temporary support device, we can fulfill these requirements successfully.

## High-strength engineering applications and temporary support systems

### Hydraulic drive high-strength temporary support system (HD-HSTSS)

**Figure 21:**
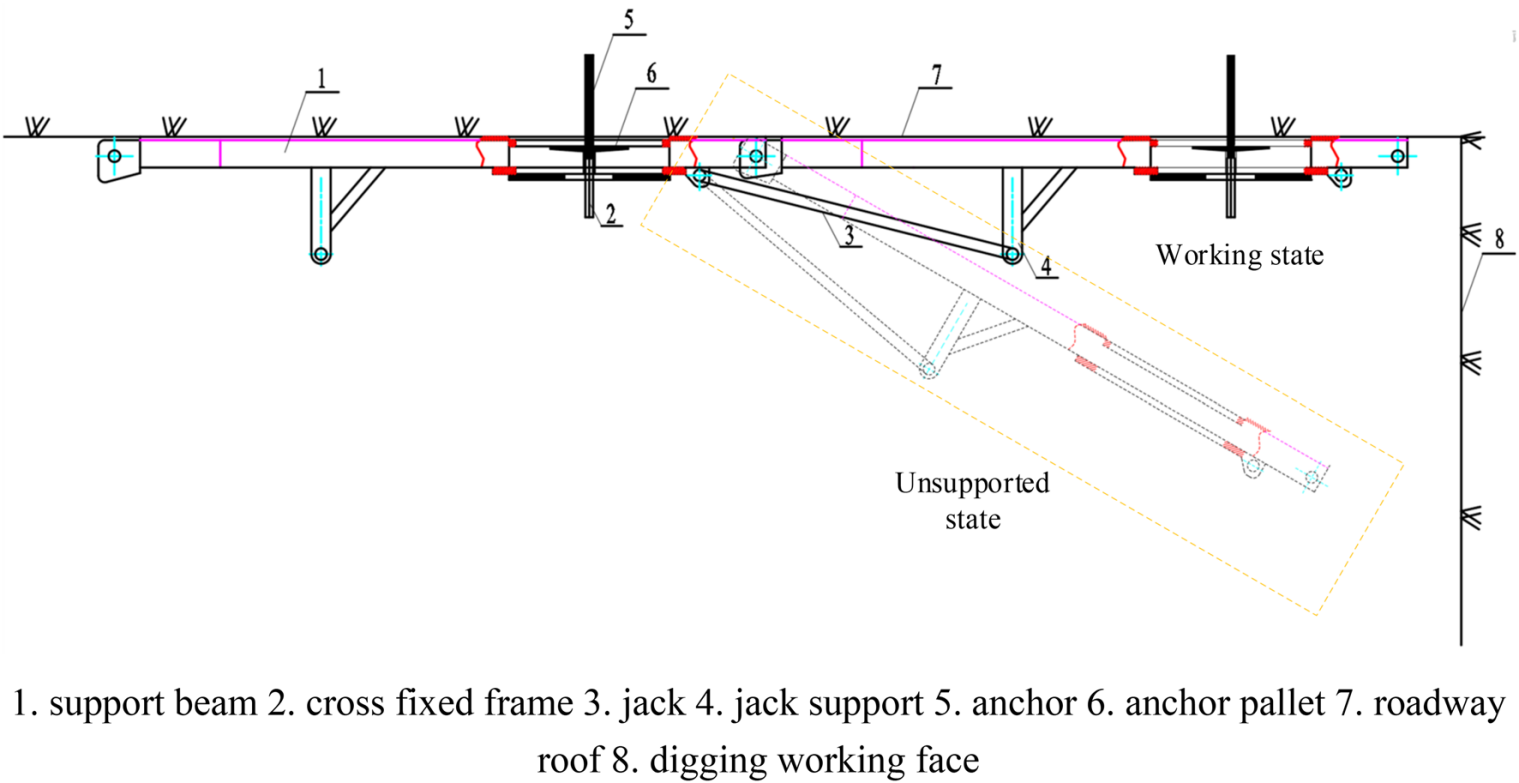
Temporary support device design drawing.

As shown in Fig. 21, the device comprises several components, such as a front probe support beam, a support stabilizer, a jack, and a cross-fixing frame. The length and number of support beams are specifically designed to meet the construction requirements of the site. The front probe support beam can be divided into sections to extend forward along the temporary support area of the road using the coupling jacks at both ends. A jack is used to support the support beam that holds up the roof. The front probe support beam has over-anchor pallet holes that allow anchor rods to be attached to the roof. These roof anchor rods are connected through cross brackets, which are securely fastened to the lower surface of the front probe support beam. The entire device can be easily disassembled and reassembled, and the front section can be adjusted to align with the direction of the roadway roof and fit tightly against it.

The detailed steps for the installation of the hydraulically operated active temporary support device are as follows:

- The first step to install the support beam, take the row of anchor rods closest to the digging head in the permanent support area as the fixing point, and install the support beam along the roadway direction, the beam body is attached to the roof plate, install the cross fixing bracket, and use the threaded sleeve to twist the end threads of the anchor rods, at this time, the support beam is installed.
- The second step is to install the jack, connect the beam and the jack with the pin, and at the same time the pin connects the beam the jack, insert the pin plate to connect the beam, at this time the jack is in the contracted state, the beam is about 60° down, and the metal mesh is laid on the beam.
- The third step to install the support beam, to the jack liquid injection, the liquid inlet port of the high-pressure ball valve closed, will be inlet pipe to the liquid inlet, open the liquid injection gun open attention to the liquid, high-pressure emulsion through the check valve into the cylinder, the jack work to promote the stent, the stent is in place after pulling out the liquid injection gun, the check valve automatically locks the cylinder high-pressure cavity; the same reason to repeat the above steps can be installed to support the beam.

The length of each beam is 800 mm, the width is 50 mm, the thickness is 10 mm and the cross hole diameter is 200 mm.

**Figure 22:**
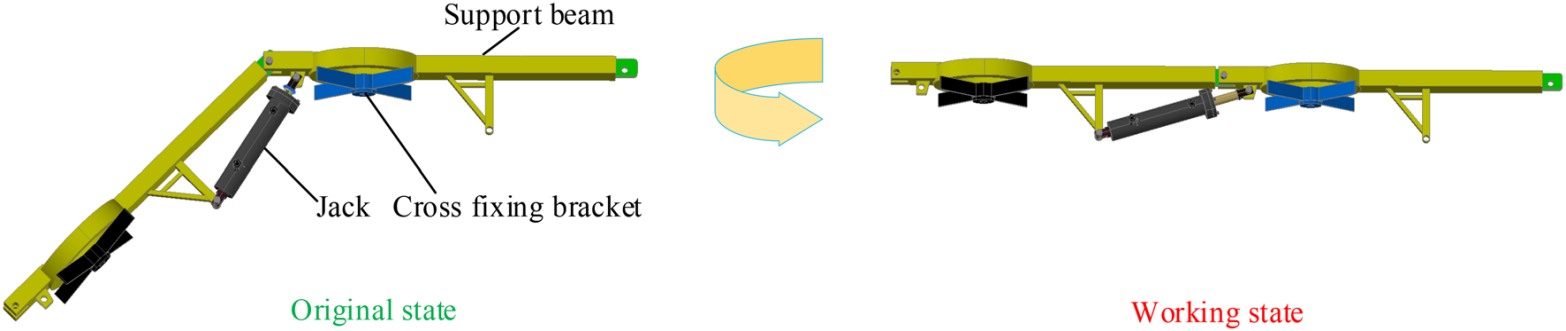
A HD-HSTSS device’s working diagram.

To attain temporary support with high strength using hydraulic power, we utilize a lateral jack. This jack provides the necessary strength for the roof, ensuring that it meets the requirements for the active support force. Figure 22 displays the working schematic of the HD-HSTSS device.

### Optimization of the temporary support process

The process of constructing roadways beneath coal mines involves several steps. First, the coal rock is broken. Then, temporary support is provided to ensure safety during construction. This temporary support includes three groups of support beams, with five anchors per row. The A4027 return airway, which has a width of 4.4 m, follows this process.

Next, the roadway machine is stopped and locked. The gun head guard is covered to create an operation platform for providing temporary support. Once the temporary support is in place, permanent support is installed in the same area. This ensures the long-term stability of the roadway.

After the permanent support operation is completed, the hydraulic-driven, high-strength temporary support devices are removed. This allows the roadway process to restart.

### Evaluation of field application and impact

**Figure 23:**
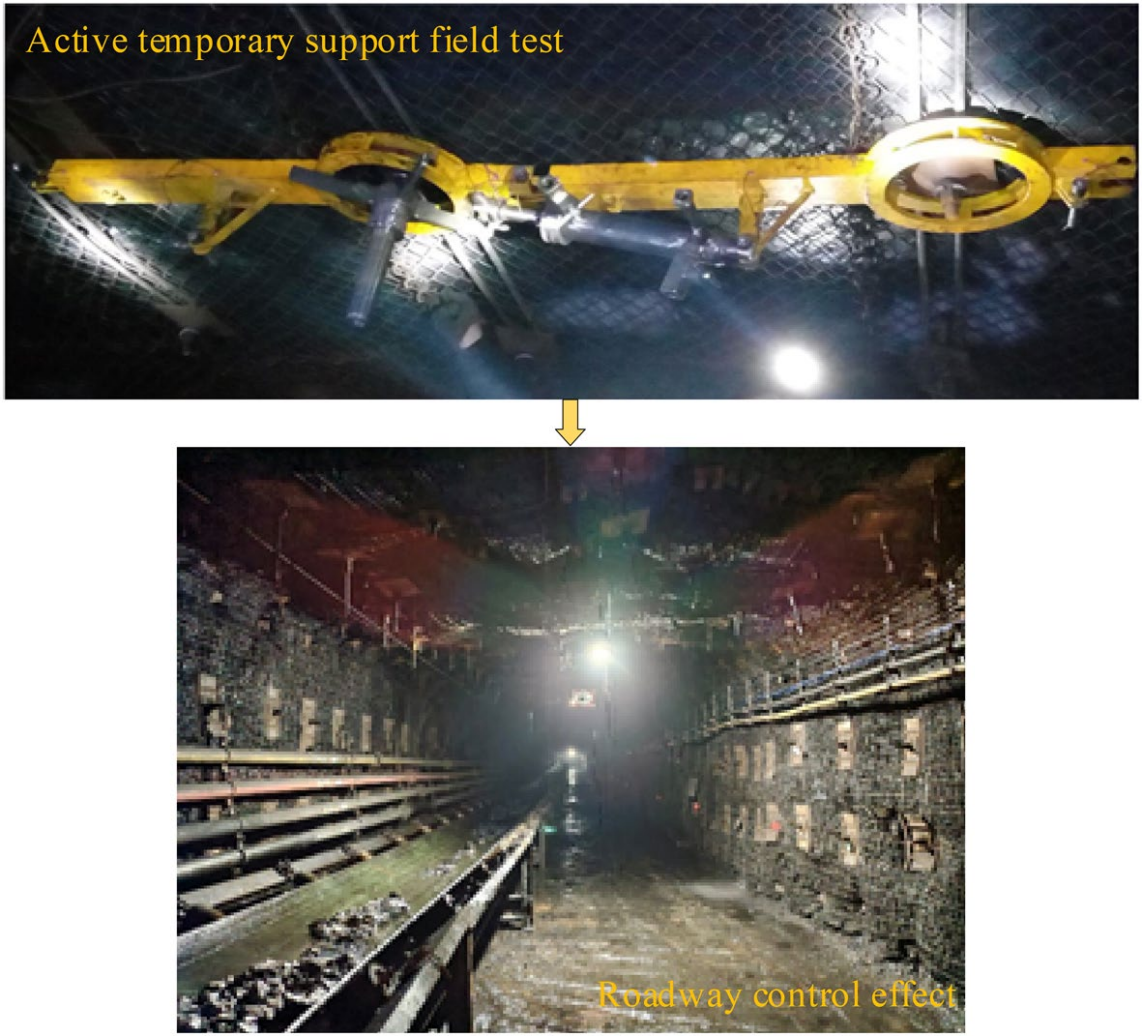
Site effect.

During the construction of the A4027 return airway, the effectiveness of the hydraulically powered, high-strength temporary support device was tested. Figure 23 illustrates the field utilization of the device during the boring process and highlights the impact of the support provided during the final stage of roadway boring.

**Figure 24:**
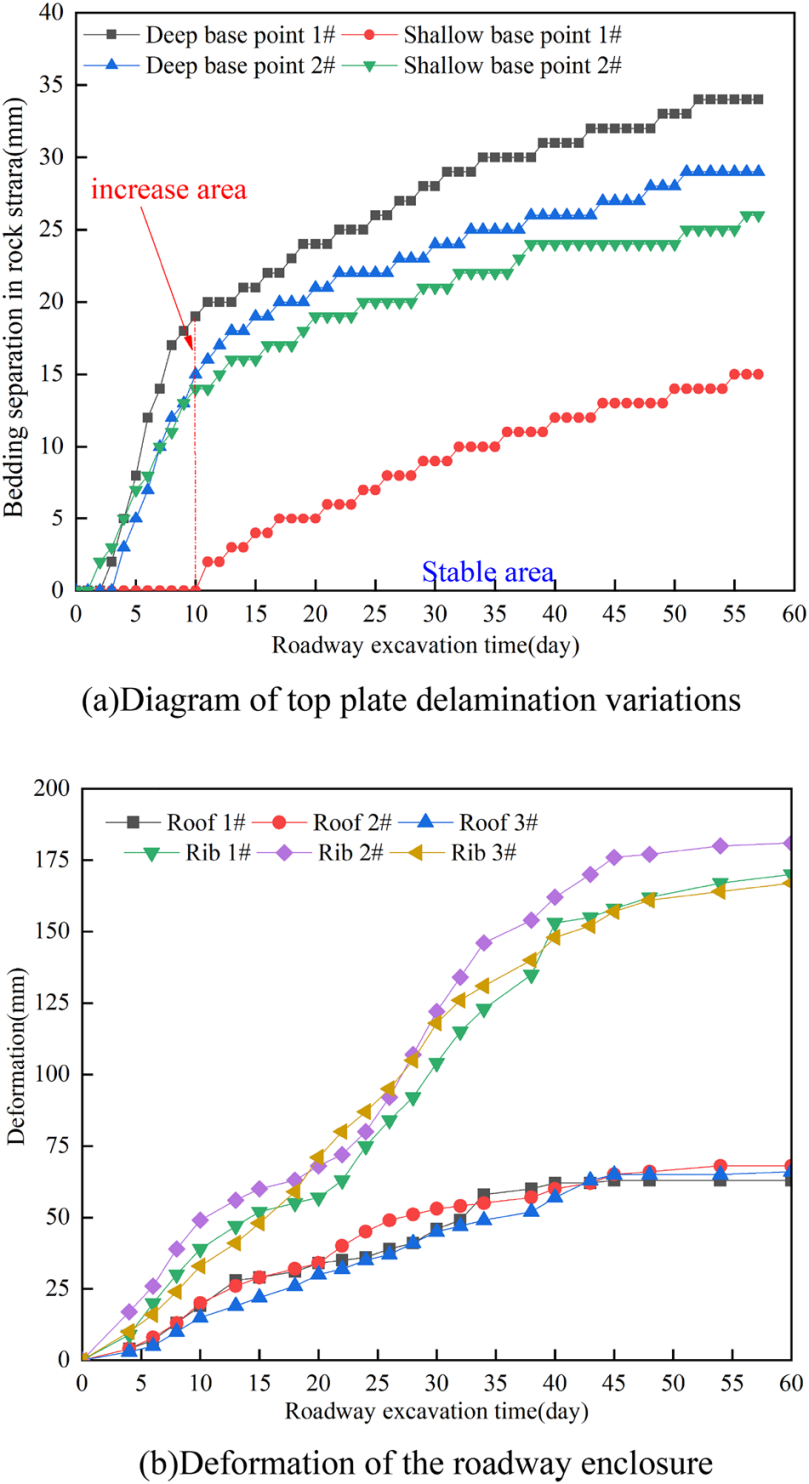
Monitoring of mine pressure on-site.

In Fig. 24, the on-site monitoring of mine pressure is depicted. During a 2-month observation period, the convergence rate in the test area of the A4027 return airway remained very low. The deepest part had a maximum convergence value of 34 mm, while the shallowest part had a maximum convergence value of 26 mm. The maximum displacements of the two gangs did not exceed 181 mm. The sinking in the roof plate was less than 68 mm, indicating effective control over the surrounding rock of the roadway.

It is important to note that the top plate of the A4027 return airway showed significant delamination changes 10 days after excavation, with no occurrences during the first 3 days. However, it gradually stabilized afterward. The overall rate of top plate deformation was low, suggesting that the use of high-strength hydraulic-driven temporary support devices improved control over top plate delamination by reducing the strength of the support system.

## Discussion

In the case of extremely soft rock formations, the traditional simple temporary support method is ineffective. It is important to note that temporary support is even more important than permanent support in these conditions. Conventional temporary shoring methods cannot provide active support, which leads to significant roof break-ups during the early stages of excavation, especially in soft-rock roadways.

To overcome these challenges, a hydraulically driven active temporary shoring system has been introduced. This support system is based on the original forebay beam and later machine-mounted temporary support, as mentioned in references^10–13^. Compared to traditional methods, hydraulically driven support offers higher force, increased flexibility due to its lightweight structure, and greater reliability due to its high structural rigidity. Most importantly, this type of temporary support plays a crucial role in controlling the initial excavation of roadways, effectively reducing surrounding rock deformation. As a result, this study has successfully ensured the stability of the surrounding rock on roadways located within highly soft rock layers.

Temporary support plays a crucial role in ensuring the stability and safety of roadways. However, existing research primarily focuses on the strength of temporary supports and the control of temporary hollow jacks. There is a lack of studies that explore the relationship between support strength and distance. In our study, we examined the use of longitudinally mounted jacks for active support. Our objective was to determine the thrust force generated by these jacks, assess their load-carrying capacity, and evaluate their effectiveness in temporary support applications. It is essential to ensure that the load-bearing capacity of the jacks does not exceed the structural limits of the surrounding members. To achieve this, engineering judgment and calculations based on structural analysis should be utilized to ensure the overall stability and integrity of the temporary support system.

Furthermore, we quantified the relationship between temporary support strength (q), support distance (L), and the ease of separation parameter (Q). It is important to acknowledge that our study has limitations, such as assumptions about idealized conditions and the exclusion of other external factors that may impact the performance of the jacks. Future research should aim to validate these findings through experimental tests and field measurements.

In conclusion, using hydraulically actuated jacks for active temporary support is a practical solution for roadways. Calculating the thrust forces provides valuable insights into the load-bearing capacity of these jacks, helping with temporary support design decisions. Implementing hydraulically driven active temporary support effectively addresses the challenge of significant initial departures and severe deformation in soft rock conditions. The successful use of HD-HSTSS on the A4027 roadway demonstrates the effective control of roadway deformation during the early excavation stages. This helps minimize damaging deformation in the permanent support system of the roadway, resulting in impressive outcomes.

## Conclusion

1. A mechanical analysis model was developed to study the stacked roof in the temporary support area. Critical conditions for roof delamination were determined, and a quantitative relationship between the critical conditions of delamination and the surrounding rock at the digging face was established. Additionally, the deformation and damage characteristics of the surrounding rock at the digging face were also analyzed.
2. The temporary support strength (q) of the A4027 return airway was determined to be 10 kN/m^2^, and the temporary support distance (L) was set at 2 m. This comprehensive determination enabled the establishment of a relationship between the temporary support strength and length and the deformation and stress of the roof and surrounding rock in the temporary support area.
3. A hydraulically driven, high-strength temporary support device has been developed and subjected to both strength tests and field tests. The tests have demonstrated successful performance, particularly in the temporary support area. The device has effectively controlled the initial separation of the top plate, with the sinking amount of the top plate not exceeding 68 mm. The displacement of the two support beams has also been limited to a maximum of 181 mm. Furthermore, during the excavation process, the maximum observed value of top plate separation was only 34 mm.

## Data Availability

The datasets used and/or analyzed during the current study are available from the corresponding author upon reasonable request.
